# Genetic contributions to two special factors of neuroticism are associated with affluence, higher intelligence, better health, and longer life

**DOI:** 10.1038/s41380-019-0387-3

**Published:** 2019-03-13

**Authors:** W. David Hill, Alexander Weiss, David C. Liewald, Gail Davies, David J. Porteous, Caroline Hayward, Andrew M. McIntosh, Catharine R. Gale, Ian J. Deary

**Affiliations:** 1 Centre for Cognitive Ageing and Cognitive Epidemiology, University of Edinburgh, 7 George Square, Edinburgh, EH8 9JZ UK; 2 School of Philosophy, Psychology and Language Sciences, Department of Psychology, University of Edinburgh, 7 George Square, Edinburgh, EH8 9JZ UK; 3 Centre for Genomic and Experimental Medicine, Institute of Genetics & Molecular Medicine, University of Edinburgh, Western General Hospital, Edinburgh, EH4 2XU United Kingdom; 4 MRC Human Genetics Unit, MRC Institute of Genetics and Molecular Medicine, University of Edinburgh, Western General Hospital, Edinburgh, EH4 2XU United Kingdom; 5 Division of Psychiatry, University of Edinburgh, Royal Edinburgh Hospital, Edinburgh, EH10 5HF United Kingdom; 6 MRC Lifecourse Epidemiology Unit, University of Southampton, Southampton, UK

**Keywords:** Psychology, Psychiatric disorders, Genetics

## Abstract

Higher scores on the personality trait of neuroticism, the tendency to experience negative emotions, are associated with worse mental and physical health. Studies examining links between neuroticism and health typically operationalize neuroticism by summing the items from a neuroticism scale. However, neuroticism is made up of multiple heterogeneous facets, each contributing to the effect of neuroticism as a whole. A recent study showed that a 12-item neuroticism scale described one broad trait of general neuroticism and two special factors, one characterizing the extent to which people worry and feel vulnerable, and the other characterizing the extent to which people are anxious and tense. This study also found that, although individuals who were higher on general neuroticism lived shorter lives, individuals whose neuroticism was characterized by worry and vulnerability lived longer lives. Here, we examine the genetic contributions to the two special factors of neuroticism—anxiety/tension and worry/vulnerability—and how they contrast with that of general neuroticism. First, we show that, whereas the polygenic load for neuroticism is associated with the genetic risk of coronary artery disease, lower intelligence, lower socioeconomic status (SES), and poorer self-rated health, the genetic variants associated with high levels of anxiety/tension, and high levels of worry/vulnerability are associated with genetic variants linked to higher SES, higher intelligence, better self-rated health, and longer life. Second, we identify genetic variants that are uniquely associated with these protective aspects of neuroticism. Finally, we show that different neurological pathways are linked to each of these neuroticism phenotypes.

## Introduction

Neuroticism is one of the five higher-order factors of personality, and is consistently identified in dimensional models of personality [1]. Neuroticism largely describes the tendency to experience negative emotions, and individual differences in neuroticism show moderate to high stability across much of the adult life course [2]. Higher neuroticism is associated with a greater risk of psychiatric disorders [3–5], and some studies link higher levels of neuroticism to an increase in all-cause mortality [6]. High neuroticism has been estimated to have an economic burden to society greater than that of substance abuse, mood disorders, or anxiety disorders [7].

Like other traits, such as height [8] or intelligence [9], neuroticism is heritable [10]. Twin and family estimates indicate that around 48% of phenotypic variance can be explained by genetic effects [11]. A subset (summing to a h^2^_SNP_ of 4–15% [10, 12]) of this heritability can be traced to genetic variants in linkage disequilibrium (LD) with genotyped common single nucleotide polymorphisms (SNPs), using genomic-relatedness-matrix restricted maximum likelihood single component (GREML-SC) analyses. However, heritability estimates derived using GREML-SC represent a lower bound for the genetic effects that can be measured using SNP data. This was shown for neuroticism by Hill et al. [10] who found that, by including the variance explained by genetic variants in poor LD with genotyped SNPs, using GREML-KIN [13], heritability from DNA analysis increased to 30%. Despite the large contributions to phenotypic variance that rarer variants and non-SNP genetic variation appear to make to individual differences in neuroticism [10], it is the common SNPs that appear to act across neuroticism, psychiatric disorders, and physical disease [14]. Understanding the common genetic contributions to neuroticism, therefore, has the potential to offer insight into the causes of psychiatric disorders, health, and longevity.

Genome-wide association studies (GWAS) have begun to identify loci that are associated with neuroticism [15, 16], with well powered replication studies also being conducted [17], as well as finding loci that display a shared association between neuroticism and psychiatric disorders [18]. However, these studies derive a neuroticism variable by summing scores on all the items of a neuroticism scale. This scoring method treats each item in the neuroticism scale equally, and error variance will also be included in the score, potentially resulting in a reduction of power in an analysis. Furthermore, should this sum-score method be used on a subset of neuroticism scale items to try and derive scores that correspond to specific aspects, or “clusters”, of neuroticism [19], each of the scores will be highly correlated with one another because of their associations with the general factor of neuroticism [20]. Consequently, it is impossible to determine whether a link between an outcome and a sum score, representing a different aspect of neuroticism, reflect associations between the outcomes and that specific aspect, or between the outcome and the general factor of neuroticism [20].

Hierarchical clustering analysis has been applied to genetic correlations between the neuroticism items in the Short-scale Eysenck Personality Questionnaire-Revised [21], in an attempt to identify genetically distinct clusters of neuroticism. This technique was used to identify groups of items that showed stronger genetic correlations with each other than with other items [19]. Cluster scores were then derived by summing the scores from these genetically more similar items to form one phenotype called “Worry” (consisting of the items “Would you call yourself a nervous person?”, “Are you a worrier?”, “Would you call yourself tense or ‘highly strung’?”, and “Do you suffer from ‘nerves’?”) and a second phenotype called “Depressed affect” (consisting the items “Do you often feel lonely?”, “Do you ever feel ‘just miserable’ for no reason?”, “Does your mood often go up and down?”, and “Do you often feel ‘fed-up’?”) [19]. However, due to the presence of a strong general factor of neuroticism in the Short-scale Eysenck Personality Questionnaire-Revised (Supplementary Table 1a & Supplementary Table 1b), sum scores will be correlated with one another and with the general factor. The result of such contamination is that the clusters, derived to be genetically homogenous, will still necessarily show strong genetic correlations with a sum-score derived general neuroticism variable, due to the variance shared across the 12 items, as can be seen in Nagel et al. [19].

**Fig. 1:**
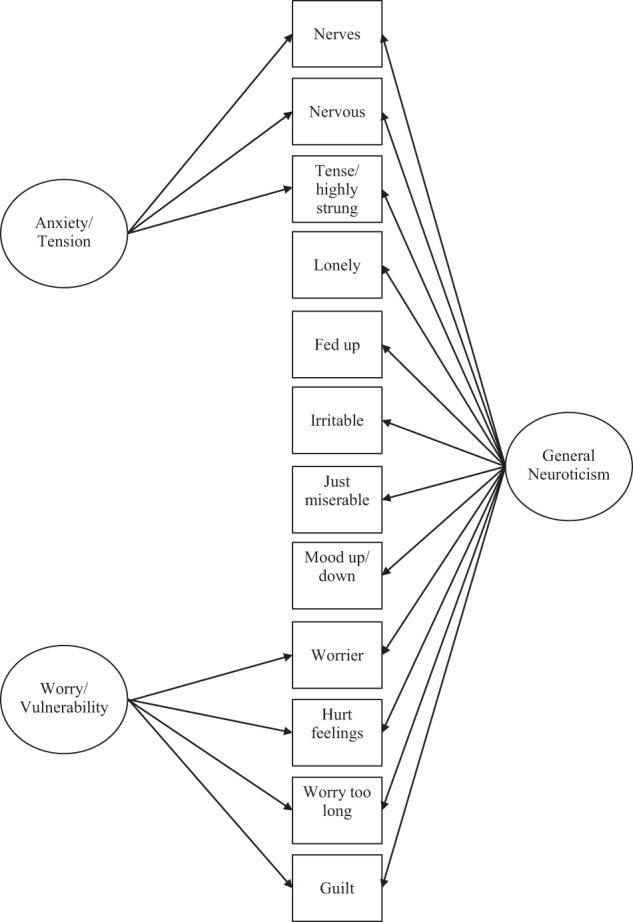
Illustrates a bifactor model applied to the 12 neuroticism items. As can be seen the use of a bifactor model results in variance that was common across the 12 items being extracted to form a general factor of neuroticism, labelled “General Neuroticism” here. Two special factors were also identified that were unrelated to the variance allocated to the general factor of neuroticism. These two special factors are labelled “Anxiety/Tension” and “Worry/Vulnerability”. Thus, the advantage afforded through the use of a bifactor model over the sum-score method such as those used by Nagel et al. [19] is that additional factors will not contain variance that is common across each of the 12 items. This allows for the discovery of the genetic associations with special (non-general) neuroticism factors that might be distinct from those with the general factor. The figure shows only those items that load most strongly on the Anxiety/Tension and Worry/Vulnerability special factors. Path coefficients for the bifactor model are shown in Supplementary Table 1a

In the present study, rather than using hierarchical clustering analysis and operationalizing the clusters as sum scores [19], we used a latent variable method—a bifactor exploratory structural equation model [22] (Supplementary Table 1a & Supplementary Table 1b) shown in Fig. 1—to identify a general factor, and two special factors of neuroticism for 401,674 UK Biobank participants who completed all 12 neuroticism items from the Short-scale Eysenck Personality Questionnaire-Revised [21]. In contrast to summing the scores from subsets of items to represent a neuroticism phenotype, a bifactor model extracts a single general factor from the variance that is common across all the items, as well as extracting additional factors that represent the degree to which the data depart from what would be expected if only a single general factor were present (Fig. 1) [22, 23]. Consequently, unlike Nagel et al.’s [19] sum scores, the special (non-general) neuroticism factors from a bifactor model are not ‘contaminated’ with variance that is common across all items.

Gale et al. previously used a bifactor model and identified two special neuroticism factors—worry/vulnerability and anxiety/tension—from the 12 neuroticism items making up the Short-scale Eysenck Personality Questionnaire-Revised [6]. They estimated factor scores to represent these special neuroticism factors and the general neuroticism factor, which sometimes had different associations with mortality: higher scores on the general neuroticism factor were associated with increased mortality from all causes and higher scores on the worry/vulnerability special factor were related to significantly decreased mortality from all causes [6].

In the current study, we sought to further this work by examining the genetic contributions to the worry/vulnerability and anxiety/tension special factors of neuroticism that Gale et al. identified previously [6]. We examine whether these special factors contrast with the genetic aetiology of a general factor of neuroticism, with which they are uncorrelated, and how the general factor and two special factors overlap genetically with mental and physical health.

## Method

### Samples

Participants were members of the UK Biobank study (http://www.ukbiobank.ac.uk) [24]. UK Biobank consists of 502,655 participants who were recruited between the years of 2006 and 2010 from the United Kingdom and were between the ages of 39 and 73 years (mean 56.9, SD = 8.0 years). Each participant provided detailed information pertaining to their background, lifestyle, and took part in cognitive and physical testing; blood, urine, and saliva samples were also provided and stored. The Research Ethics Committee granted ethical approval for the study—reference 11/NW/0382—and the current analyses were conducted under data application 10279.

### Phenotype measurement

Neuroticism was assessed using the 12 questions (e.g., “Does your mood often go up and down?” all items can be found in Supplementary Table 1) from the Short-scale Eysenck Personality Questionnaire-Revised [21]. UK Biobank participants were administered these items using touchscreens and were instructed to “Work quickly and do not think about the exact meaning of the question.” Participants were asked to choose one of four responses for each question: “Yes” (coded 1), “No” (coded 0), “Do not know” (coded—1) and “Prefer not to answer” (coded—3). Of the 502,655 UK Biobank participants, 100,981 did not answer “Yes” or “No” to all 12 questions, and so were excluded from further analyses.

### Genotyping and quality control

Details of the procedures used in the genotyping of the UK Biobank are available elsewhere [25]. In brief, two custom genotyping arrays were used to genotype 49,950 participants (UK BiLEVE Axiom Array) and 438,427 participants (UK Biobank Axiom Array), respectively [25, 26]. Genotype data on 805,426 markers were available for 488,377 of the participants in UK Biobank. Imputation was performed using a combination of the Haplotype Reference Consortium (HRC) reference panel, 1000 genomes, and UK10K. As advised by UK Biobank, we restricted the imputation analysis to the HRC panel. This led to 39,131,578 autosomal SNPs, after imputation, being available for the 270,059 participants who had completed all 12 neuroticism items [25]. Allele frequency checks [27] were performed against the HRC [28] and 1000G [29] site lists, and variants were removed if the allele frequencies differed from the reference set by more than +/− 0.2.

Additional quality control was implemented in the data used in the present study, and included the removal of participants with non-British ancestry (identified by Bycroft et al. [25] by performing a principal component analysis on the genotyped SNP data to remove ethnic outliers from a subset of the UK Biobank participants who self-identified as White British) as well as those who had an extreme score based on heterozygosity (extreme scores were defined as those with a principal component-adjusted heterozygosity score above 0.19 as shown by Bycroft et al. [25]) and >5% missingness [25]. Individuals were also removed if their reported sex was inconsistent with genetically inferred sex, as were individuals with neither XX nor XY chromosomes. Finally, individuals with more than 10 putative third degree relatives (identified by Bycroft et al. [25] by estimating the kinship coefficients for all pairs of samples using the software KING [30]) were also removed. This left 408,095 individuals. Using GCTA [31] on 131,790 reportedly-related participants [31, 32] one from each pair of related individuals was removed using a genetic relationship threshold of 0.025, resulting in 332,050 individuals. Following these quality control steps, a sample size of 270,059 individuals had both genetic data and had answered all 12 neuroticism items. SNPs with a minor allele frequency (MAF) <0.0005, and an imputation quality score <0.1 were removed along with multi-allelic SNPs, resulting in 18,485,882 autosomal SNPs.

### Statistical analysis

#### Exploratory bifactor analysis of neuroticism items

For the 401,674 participants who answered “yes” or “no” to all 12 neuroticism items, like Gale et al. [6], we used Mplus version 8.2 [33] to obtain estimates of three latent bifactor scores for each participant. To do so we first conducted an exploratory bifactor analysis. This involved using exploratory structural equation modelling analysis to extract the three factors and then applying an oblique bifactor Geomin rotation to the resulting 12 × 3 factor loading matrix [22, 23].

A Geomin rotation of a *p* × *k* factor loading matrix *Λ* is one in which correlated axes are rotated to minimize the criterion:

$${\mathrm{geomin}}\left( \Lambda \right) = \mathop {\sum}\limits_{i = 1}^p {\left( {\mathop {\prod}\limits_{r = 1}^k {\left( {\lambda _{ir}^2 + \varepsilon } \right)} } \right)^{\frac{1}{k}}}$$

where *p* refers to the row, *k* refers to the column, *λ* is the loading of a factor on an item, and ε = 0.01 [34]. The criterion is thus at its minimum when every item loads on one single factor. In the bifactor case, this criterion is applied only to the last *k*-1 factors, so that the criterion is minimized when each item loads on only one of these factors.

The bifactor model showed evidence of good model fit: the root mean square error of approximation was 0.048 (90% CI = 0.048–0.049), the comparative fit index was 0.975, the Tucker Lewis index was 0.951, and the standardized root mean square residual was 0.019. The factor loadings are presented in Supplementary Table 1a and Supplementary Table 1b (The correlation matrix can be found in Supplementary File 1). The first factor, which explained 29% of the variance, was a general factor of neuroticism onto which all 12 items loaded. The second and third factors extracted were special factors and so are uncorrelated with the general factor. The second factor was characterized by loadings on the items in the neuroticism scale of “Would you call yourself a nervous person?” (loading of 0.60), “Do you suffer from ‘nerves’?” (loading 0.49), and “Would you call yourself tense or ‘highly strung’?” (loading of 0.35). Due to each of these neuroticism items describing feeling tense or anxious we called this residual variance the anxiety/tension special factor of neuroticism. As special factors are uncorrelated with the variance in the general factor of neuroticism the special factor of anxiety/tension can be considered what is left of anxiety/tension following the removal of the general factor of neuroticism. The third factor was characterized by loadings on the items in the neuroticism items of “Do you worry too long after an embarrassing experience?” (loading of 0.57), “Are your feelings easily hurt?” (loading of 0.40), “Are you a worrier?” (loading of 0.31), and “Are you often troubled by feelings of guilt?” (loading of 0.31). As each of these neuroticism items describes feelings of being vulnerable or ruminating and worrying, we called the residual variance that is common across these three items the worry/vulnerability special factor of neuroticism. This special factor of worry/vulnerability can be considered what is left of worry/vulnerability following the removal of the general factor of neuroticism. The correlations between the general factor and each of the two special factors was defined as zero in the model; the correlation between the two special factors in the model was r = 0.311 (SE = 0.006, 95% CI = [0.301, 0.321], *P* < 0.0001). As in Gale et al. [6], the relationship between these two aspects of neuroticism was not attributable to variance from the general factor of neuroticism, as indicated by a correlation of zero between each of the special factors with the general factor [35].

The special factors accounted for 7 and 6% of the variance, respectively, and their salient loadings, that is those greater than 0.3, ranged from 0.310 to 0.604 (median = 0.403). These special factors were thus modestly well defined. A prior analysis by Weiss et al. (2019) [36] found that the general factor and both the anxiety/tension and worry/vulnerability special factors clearly replicated (congruence coefficients = 1.00, 1.00, and 0.98, respectively) in the 1,434 participants used to develop the Short-scale Eysenck Personality Questionnaire-Revised [21]. Weiss et al. [36] also found that these factors mostly replicated in a subsample of 8,158 participants from the Generation Scotland: Scottish Family Health Study (GS:SFHS) cohort [37, 38] (congruence coefficients = 0.99, 0.93, and 0.99, respectively). Of note is that the subsample of GS:SFHS used in Weiss et al. [36] is of greater size than the unrelated sample used in the current study to conduct the genetic analysis.

#### Genome-wide association analysis (GWAS) in the UK Biobank sample

The score on the general factor and both special factors were adjusted for age, sex, assessment centre, genotype batch, array, and 40 genetic principal components derived from genotyped SNPs. Association analysis was performed separately for each of the three neuroticism phenotypes using an additive model implemented using BGENIE [25].

#### Linkage disequilibrium score regression

Univariate Linkage disequilibrium score (LDSC) regression [39] was used to derive a SNP-based heritability estimate for each of the three neuroticism factors, as well as to quantify the amount of residual stratification in each data set. Bivariate LDSC regression was used to derive genetic correlations [40] with other phenotypes.

Genetic correlations derived using LDSC are informative of the shared genetic aetiology between two traits. They can be interpreted as the degree to which the heritable variation of two traits is correlated, and so do not describe the absolute magnitude of the effect on the phenotype created by this overlap. For example, two traits could have a very low, but non-zero, heritability, and still show a very high genetic correlation. In this instance, the interpretation would be that, whereas genetic effects explain only a negligible portion of phenotypic variance for each trait, as evidenced by the low heritability of each trait, the genetic effects that explain variance in one trait, are largely the genetic effects that explain phenotypic variance in the second trait, as evidenced by the high genetic correlation between the two hypothetical traits. In addition, providing the phenotypic correlation between two traits is not zero, the magnitude of the phenotypic correlation between traits is not an indicator of the magnitude of the genetic correlation between the same traits. For example, two traits may have a very low phenotypic correlation with each other but have a very high genetic correlation. For example, the genetic correlations between the 12 items of the Short-scale Eysenck Personality Questionnaire-Revised [21] in Nagel et al. (2018) [19] are far larger than their phenotypic correlations. In this instance, the interpretation would be that, whereas these traits share very little variance with each other, as evidenced by the low correlation, the variance that is genetic in origin is largely shared across these traits, as evidenced by the large genetic correlation.

A MAF cut-off of <0.01 was applied. Only SNPs that were in HapMap 3 with MAF >0.05 in the 1000 Genomes EUR reference sample were included. Next, indels and structural variants were removed as were strand ambiguous variants. SNPs whose alleles did not match those in the 1000 Genomes were also removed. The presence of outliers can increase the standard error in LD score regression [40], and so SNPs with a χ^2^ > 80 were removed. LD scores and weights for use with European populations were downloaded from (http://www.broadinstitute.org/~bulik/eur_ldscores/). Because we tested 32 genetic correlations (Alzheimer’s disease was included twice) against each of the three neuroticism factors, we controlled for the false discovery rate (FDR) using the Benjamini–Hochberg [41] procedure. Full details of each of these GWAS along with links (where possible) to the data used can be found in Supplementary Table 2.

#### Identification of independent genomic loci and functional annotation

Genetic loci related to each of the three factors of neuroticism were identified using Functional Mapping and annotation of genome-wide association studies (FUMA, http://fuma.ctglab.nl/) First, independent significant SNPs were identified on the basis of their *P*-value being genome-wide significant (*P* < 5 × 10^−8^), and being independent from each other (r^2^ < 0.6) within a 1mb window. Second, SNPs that were in LD with the independent lead SNPs (r^2^ ≥ 0.6) within a 1mb window, and in the 1000 genomes reference panel with a MAF greater than 0.0005, were included for further annotation. Third, lead SNPs were identified using the independent significant SNPs defined as above. Lead SNPs were a subset of the independent significant SNPs that were in LD with each other at r^2^ < 0.1, again with a 1mb window. Fourth, genomic risk loci were identified by merging lead SNPs if they were closer than 250 kb apart, meaning that a genomic risk locus could contain multiple independent significant SNPs and multiple lead SNPs. Finally, all SNPs in LD of r^2^ ≥ 0.6 with one of the independent significant SNPs formed the border, or edge, of the genomic risk loci. To map LD, the 1000 genomes phase 3 was used [42].

Functional annotation of each of the three neuroticism factors was carried out in FUMA (http://fuma.ctglab.nl/) using all SNPs found within the independent genomic loci that were in LD of r^2^ ≥ 0.6, were nominally significant, and had a MAF of ≥0.0005. To assess the functional consequences of genetic variation at these SNPs, they were first matched based on chromosome, base pair position, reference, and non-reference alleles to a database containing functional annotations including the Annotate Variation (ANNOVAR) categories [43], Combined Annotation Dependent Depletion (CADD) scores [44], Regulome Database (RDB) scores [45], and chromatin states [46–48].

ANNOVAR [43] categories were used to identify the function of the SNP and to locate their position. A CADD score is a continuous measure that is used to assess how deleterious genetic variation at the SNP is to protein structure and function. Higher CADD scores are indicative of a more deleterious variant, with scores of greater than 12.37 providing evidence of pathogenicity [44]. RDB scores are categorical measures created using data from expression quantitative trait loci (eQTLs) as well as chromatin marks. RDB scores range from 1a to 7 with lower scores indicating greater evidence for the variant having a regulatory function.

Chromatin states can be used to predict transcription/regulatory effects at SNP loci. This was described using a 15-point scale for each variant using a hidden Markov model based on five chromatin marks for 127 epigenomes in the Roadmap Epigenomics Project [47]. The lower the chromatin score the greater the level of accessibility to the genome at this site, indicating open chromatin sites, with scores of less than 8 indicative of an open chromatin region. The minimum chromatic state across tissues was used.

#### Gene-mapping

Three techniques were used to link the association found in the independent genomic loci to genes. First, SNPs were mapped to genes based on physical distance. For this positional mapping technique, SNPs within a 10kb window from the known protein genes found in the human reference assembly (hg19) were included.

Second, eQTL mapping was carried out. Here, SNPs were mapped to genes if allelic variation at the SNP is associated with expression levels of the gene. For eQTL mapping, information on 45 tissue types from three data bases (GTEx, Blood eQTL browser, and BIOS QTL browser) based on cis-QTLs where SNPs are mapped to genes up to 1Mb away. An FDR of 0.05 was used as a cut-off to define significant eQTL associations.

Finally, chromatin interaction mapping was performed. SNPs were mapped to genes if there was a three-dimensional DNA–DNA interaction between the SNP region indicated by the independent genomic loci, with the gene region. No distance boundary was used as chromatin interactions can involve long-range interactions between SNPs with genes. Hi-C data of 14 tissue types were used for chromatin interaction mapping [49]. Chromatin interactions can span multiple genes, due to SNPs being located in regions that interact with other regions that also contain multiple genes. To both reduce the number of genes mapped, and to increase the likelihood that those mapped are those that are biologically relevant to the independent genomic loci, we selected only interaction mapped genes where one region involved with the interaction overlapped with a predicted enhancer region in any of the 111 tissue/cell types found in the Roadmap Epigenomics Project [47], and the other region was located in a gene promoter region (250bp upstream and 500bp downstream of the transcription start site and also predicted to be a promoter region by the Roadmap Epigenomics Project [47]). We used an FDR of 1 × 10^−5^ to define a significant interaction.

#### Gene-based association analysis

Gene-based analysis was conducted using Multi-marker Analysis of GenoMic Annotation (MAGMA) [50]. SNPs from the summary statistics from each of the three neuroticism phenotypes were matched to genes according to the NCBI 37.3 build with gene boundaries being defined as the stop and start site. To model linkage disequilibrium the reference panel from the 1000 Genomes (phase 1, release 3) was used. This led to 18,330 autosomal genes being available for analysis. To control for multiple testing, a Bonferroni correction was used resulting in an alpha level of 2.728 × 10^−6^ for the three phenotypes.

#### Gene-set analysis

Using MAGMA [50], gene-set analysis was conducted using competitive testing. A total of 10,894 gene-sets, which were sourced from Gene Ontology [51], Reactome [52], the Molecular Signatures database (MSigDB) [53], and other sources, were examined for enrichment in each of the three factors of neuroticism. A Bonferroni correction was applied to control for the multiple tests performed on the 10,894 gene sets.

#### Gene-property analysis

To examine the importance of particular tissue types relevant to the neuroticism factors, a gene-property analysis was conducted using MAGMA. This analysis was used to determine if, in 30 broad tissue types, and 53 specific tissues, tissue-specific differential expression levels were predictive of the association of a gene with each of the three factors of neuroticism. Tissue types were taken from the GTEx v7 RNA-seq database [54] with expression values being log2 transformed with a pseudocount of 1 after winsorizing at 50 with the average expression value being taken from each tissue. Multiple testing was controlled for using Bonferroni correction for 30, and 53 tests. A separate gene-property analysis was used to determine if transcription in the brain at any one of 11 developmental stages [55], or across 29 different ages [55], was associated with a gene’s link to each of the factors of neuroticism. A Bonferroni correction was used to control for 11 and 29 tests separately.

#### Genetic prediction

Genetic prediction was conducted with two goals. First, to examine if the polygenic signal associated with the general factor of neuroticism, and each of the two special factors, replicated in an independent sample. Second, to assess if unlike the general factor of neuroticism, those with a greater polygenic burden for higher levels of the special factor of anxiety/tension, and the special factor of worry/vulnerability, have greater level of phenotypic income and intelligence, as would be expected from the genetic correlation analysis. Polygenic risk scores (PGRS) were derived using PRSice-2 [56] (https://choishingwan.github.io/PRSice/) and the GS:SFHS cohort [37, 38].

The recruitment protocol and sample characteristics of GS:SFHS are described in full elsewhere [37, 38]. In brief for GS:SFHS, 23,690 participants were recruited through their GP from across Scotland. Participants were all aged 18 and over and were not ascertained based on the presence of any specific disease.

Neuroticism was measured in GS:SFHS using the Short-scale Eysenck Personality Questionnaire-Revised [21]. Participants were omitted if they did not answer one or more of the 12 neuroticism items, or if they had also taken part in UK Biobank. Finally, participants were removed from the analysis in the event that they were related to another member of GS:SFHS at 0.025 as ascertained using a genomic relationship matrix derived using GCTA [31]. For all phenotypes examined, SNPs were included in the data if they had a MAF ≥ 0.01 and Hardy-Weinberg *P*-value of 0.000001 and individuals who had taken part in UK Biobank were also removed from the GS:SFHS data set (*n* = 174).

As conducted in UK Biobank and Gale et al. [6] for the remaining 6907 participants of GS:SFHS, Mplus version 8.2 [33] was used to carry out an exploratory structural equation model with an oblique bifactor Geomin rotation [22, 23]. Again three scores were derived for each participant. The bifactor model for GS:SFHS is presented in Supplementary Table 1a and 1b (the correlation matrix can be found in Supplementary File 2). Using GS:SFHS there was evidence of a general factor which each of the 12 items loaded on. This factor was nearly identical to its counterpart in the structure derived from UK Biobank (congruence coefficient = 0.98).

The same second factor as identified in UK Biobank (anxiety/tension special factor of neuroticism) was also identified in GS:SFHS. This second factor was characterized by loading on the items of “Would you call yourself a nervous person?” (loading of 0.378), “Do you suffer from ‘nerves’?” (loading of 0.315), and “Would you call yourself tense or ‘highly strung’?” (loading of 0.198) from the neuroticism scale. This similarity between this factor and its counterpart in the structure derived from UK Biobank (congruence = 0.84) fell just short of the level (0.85) [57] leading one to conclude that the factors were similar.

The special factor of worry/vulnerability was also identified in GS:SFHS. This third factor was again characterized by loadings on the items in the neuroticism items of “Do you worry too long after an embarrassing experience?” (loading of 0.564), “Are your feelings easily hurt?” (loading of 0.403), “Are you a worrier?” (loading of 275), and “Are you often troubled by feelings of guilt?” (loading of 0.328). The general factor and each of the two special factors were aligned so that a greater score on each is indicative of a greater level of the general factor, the anxiety/tension special factor, and the worry/vulnerability special factor. This factor was nearly identical to its counterpart in the structure derived from UK Biobank (congruence coefficient = 0.99).

 For household income a total of 6,680 participants were available from GS:SFHS who had both phenotypic and genetic data, and who were unrelated at 0.025. Income was assessed in GS:SFHS by 6-point scale (1 less than £10,000, 2 between £10,000 and £30,000, 3 between £30,000 and £50,000, 4 between £50,000 and £70,000, 5 more than £70,000, and 6 prefer not to answer). Individuals who preferred not to answer were excluded from the analysis. For intelligence a total of 7,261 participants from GS:SFHS contributed data on a general factor of intelligence derived in the same manner as previously reported [10]. This general factor of intelligence was derived by extracting the first principal component from four cognitive tests that accounted for 42.3% of the variance in the total sample. The four tests used were the Mill Hill Vocabulary Scale, the Wechsler Digit Symbol Substitution Task, the Wechsler Logical Memory tests, and the phonemic verbal fluency test. 

Each participant’s phenotypic score was then used as a predictor in a regression analysis with age, sex, and 20 principal components included to control for population stratification in GS:SFHS. The standardized residuals from this model were then used as each participant’s phenotype. For each phenotype combination examined, PRSice-2 was used to create five PGRS corresponding to one of five *P*-value cut offs (*P* ≤ 0.01, *P* ≤ 0.05, *P* ≤ 0.1, *P* ≤ 0.5, *P* ≤ 1) applied to the association statistics from the summary data. The polygenic risk scores were then standardized and used in a regression model for phenotype prediction in GS:SFHS.

## Results

As described above, the correlation of the general factor and each of the special factors in the bifactor model was defined as 0. The correlation between the two special factors was estimated in the model as *r* = 0.311. We estimated each participant’s score on each of the three factors in the bifactor model of neuroticism. A correlation of the estimates of the latent score for the general factor scores and the anxiety/tension special factor scores in UK Biobank was found to be *r* = 0.069. A correlation between the general factor and the worry/vulnerability special factor scores was also identified in UK Biobank as *r* = 0.121, (*P* < 2.20 × 10^−16^). The special factor scores of worry/vulnerability and anxiety/tension correlated with each other at *r* = 0.427 (*P* < 2.20 × 10^−16^) in UK Biobank (Table 1). Small differences, such as these, between correlations derived using the factors within the model and correlations of the participants’ estimated scores on the latent variables are expected [58].

**Table 1:** Showing the heritability of each factor on the diagonal, with the phenotypic correlations on the lower half, and the genetic correlations on the upper half. Standard errors displayed in brackets. Heritability and genetic correlations were derived using LDSC regression

	General factor of neuroticism	Anxiety/tension special factor of neuroticism	Worry/vulnerability special factor of neuroticism
General factor of neuroticism	11% (0.5%)	0.20 (0.04)	0.35 (0.03)
Anxiety/tension special factor of neuroticism	0.07	5.7% (0.3%)	0.66 (0.02)
Worry/vulnerability special factor of neuroticism	0.12	0.43	6.4% (0.3%)

We estimated the heritability of each of the neuroticism phenotypes using univariate LDSC regression and showed a total of 10.7% (SE = 0.49%) of the phenotypic variation in the general factor of neuroticism is explained by the additive effects of common genotyped SNPs (Table 1). This is comparable to the estimate of 10.8% (SE = 0.50%) and 10.0% (SE = 0.30%) using a sum-score approach also derived using UK Biobank [15, 16]. The additive effects of common genotyped SNPs explained 5.7% (SE = 0.32%) and 6.4% (SE = 0.28%) of the variance in the anxiety/tension and worry/vulnerability special factors, respectively. The general factor of neuroticism had a genetic correlation of *r*_g_ = 0.98 (SE = 0.003, *P*  > 1 × 10^−200^) with the sum-score neuroticism [15] phenotype. The general factor was found to have a positive genetic correlation with the anxiety/tension special factor, *r*_g_ = 0.20 (SE = 0.04, *P* = 2.05 × 10^−8^), and with the worry/vulnerability special factor, *r*_g_ = 0.35, (SE = 0.03, *P* = 1.48 × 10^−32^); the two special factors had a strong positive genetic correlation with each other, *r*_g_ = 0.66, (SE = 0.02, *P* = 5.96 × 10^−187^) (Table 1).

### Loci discovery and annotation of the general factor of neuroticism and the two special factors

**Fig. 2:**
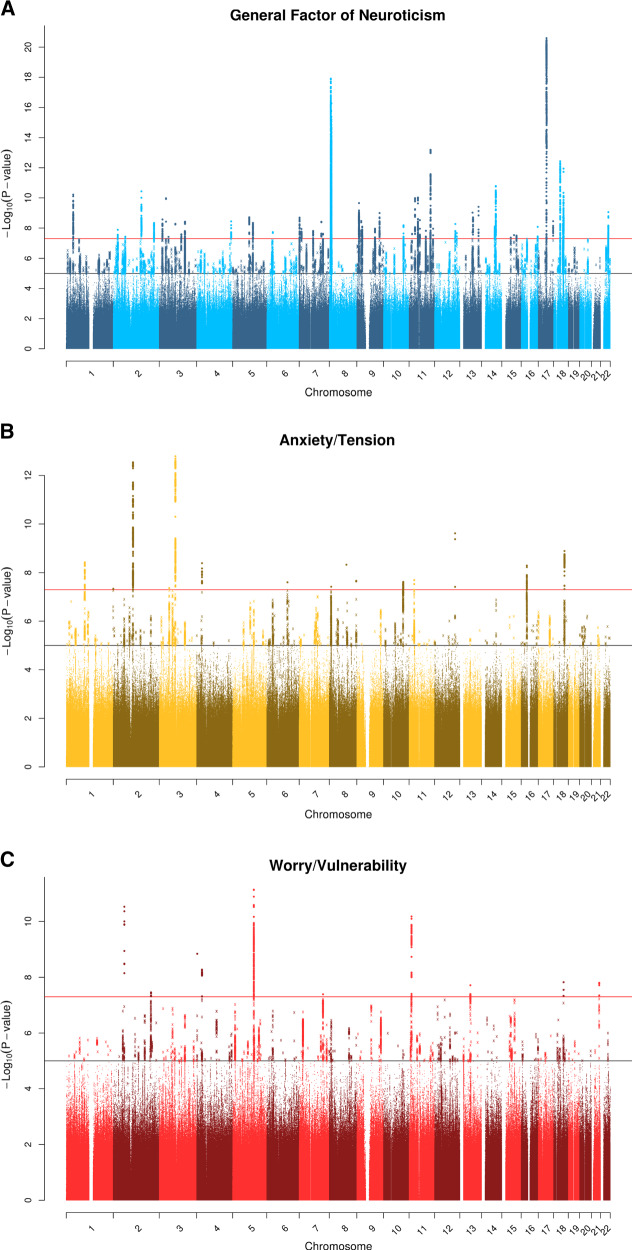
Manhattan plots for the factors of neuroticism. All sample sizes were 270,059 participants. The red line indicates genome-wide significance and the black indicates suggestive significance. Figure 2a indicates the general factor of neuroticism (Blue). Figure 2b the anxiety/tension special factor (Gold). Figure 2c the Worry/vulnerability special factor (Red)

For the general factor of neuroticism, 51 independent loci were identified as genome-wide significant (Fig. 2a, Supplementary Table 3). A total of 44 of these loci overlapped with loci identified using the data from Luciano et al. [15] analyzed using FUMA [59] with the same settings as those used in the current study (Supplementary Table 4). A total of 42 loci overlapped with those identified by Nagel et al. [17] who re-analyzed the Luciano et al. [15] data sets (Supplementary Table 4). These 51 loci were found to contain an overrepresentation of SNPs found in introns (49.69%), as well as SNPs found in intergenic regions (25.15%) with the two next largest categories being ncRNA intronic (14.67%), and ncRNA exonic (2.26%) (Supplementary Table 3). Evidence was also found that these loci contained regulatory regions of the genome, indicated by 32.50% of the SNPs in the genomic loci having RDB less than 2, indicating that genetic variation at this SNP is likely to affect gene expression (Supplementary Table 3). Finally, 86.49% of the SNPs within the genomic loci had a minimum chromatin state of <8 indicating that they are located in an open chromatin state, providing additional evidence that they are located within regulatory regions (Supplementary Table 3).

Using the GWAS catalogue, lead and tagging SNPs from the 51 loci identified for the general factor of neuroticism were examined for overlap with other traits. A total of 42 of the 51 loci were found to overlap with loci previously associated with neuroticism [15], as would be expected. Five loci also showed overlap with educational attainment. In addition, variations in these 51 loci have been associated with psychiatric disorders including, autistic spectrum disorder (four loci) and schizophrenia (eight loci) (Supplementary Table 5).

For the anxiety/tension special factor, 14 independent genomic loci were identified using a threshold of 5 × 10^−8^ (Fig. 2b, Supplementary Table 6), only one of which physically overlapped with the general factor of neuroticism and with neuroticism operationalized using the sum-score method [14] (loci 8 on chromosome 8, bp position 10576753–10732050) (Supplementary Table 7). However, four additional loci associated with the anxiety/tension special factor of neuroticism overlapped with the sum-score-derived neuroticism score used in Nagel et al.’s [17] re-analysis of the Luciano et al. [15] data sets. As with the general factor, these independent loci harbored a greater proportion of SNPs located in introns (63.0%), and intergenic regions (26.9%). Of these SNPs, 31.1% had an RDB score of less than 2 and 65.8% had a minimum chromatin value of less than 8 providing further evidence that these variants are located in regions of the genome that are linked to gene regulation (Supplementary Table 6). These 14 loci showed overlap with the loci identified in previous GWAS examining carotid intima media thickness (one locus), coronary artery disease (two loci), white matter lesion progression (one locus), systolic blood pressure (one locus), diastolic blood pressure (one locus), HDL cholesterol (two loci), adiposity traits, weight, and body mass index (five loci) (Supplementary Table 8).

A total of ten genome-wide significant loci were identified (Fig. 2c, Supplementary Table 9) for the worry/vulnerability special factor. Only one of these loci overlapped with the loci identified for the general factor of neuroticism (locus 9 on chromosome 18, bp position 53210302–53464917), two overlapped with the sum-score derived neuroticism variable used by Luciano et al., and three overlapped with the re-analysis of the Luciano et al. data sets conducted by Nagel et al. [16]. Only one locus overlapped with the anxiety/tension special factor (locus 4 on chromosome 4, bp position 28519689–28660963) (Supplementary Table 7). The SNPs in these regions were again found to be predominantly located within introns (46.5%), and intergenic regions (32.0%). Evidence that the genome-wide significant loci harboured SNPs that were linked to gene expression was found again where 31.8% of candidate SNPs had a RDB score of less than 2 and 67.0% had a minimum chromatin value of less than 8 (Supplementary Table 9**)**. Using the GWAS catalogue, these 10 independent genomic loci have previously been linked to, hand grip strength (1 locus), brain cytoarchitecture (1 locus), coronary artery disease (1 locus), and chronotype (1 locus) (Supplementary Table 10).

### Gene mapping of the general factor of neuroticism and the two special factors

**Fig. 3:**
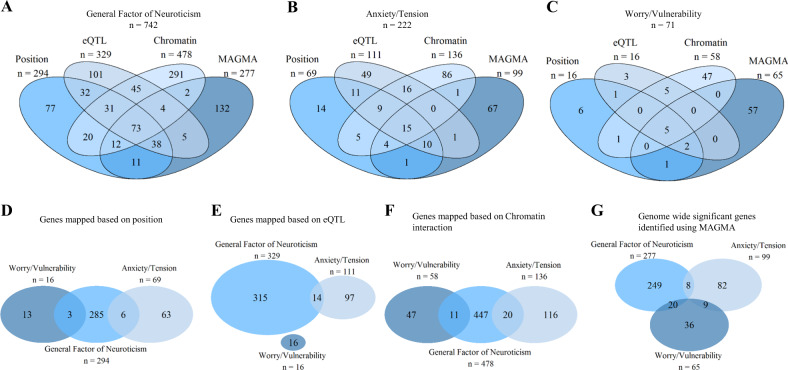
Panel **a**, **b**, and **c** show Venn diagrams illustrating the overlap of the genes indicated using positional mapping, eQTL mapping, chromatin interaction mapping, and the genome-wide significant gene-based statistics derived using MAGMA, conducted on the general factor of neuroticism (**a**), the anxiety/tension special factor (**b**), worry/vulnerability special factor (**c**). Panel **d**, **e**, **f**, and **g** use Venn diagrams to examine the overlap in the genes implicated across the three neuroticism phenotypes using positional mapping (**d**), eQTL mapping (**e**), chromatin interaction mapping (**f**), and the genome-wide significant gene-based statistics derived using MAGMA (**g**)

This section details the genes that were linked to the loci that were associated with the general factor of neuroticism and both special factors. Positional mapping aligned the SNPs from the independent genomic loci associated with the general factor of neuroticism to 294 genes by using location, whereas eQTL mapping matched cis-eQTL SNPs to 329 genes whose level of expression they have been shown to influence. Finally, chromatin interaction mapping annotated SNPs to a total of 478 genes using three-dimensional DNA–DNA interactions between the SNPs’ genomic regions and close or distant genes (Supplementary Table 11, Supplementary Figure 1). Collectively these mapping strategies identified 742 unique genes, of which 255 were implicated by two mapping strategies and 104 being implicated by all three. Three genes, *STARD6*, *C18orf54*, and *CCDC68*, implicated using chromatin mapping, showed evidence of a chromatin interaction between two independent genomic risk loci (Supplementary Table 12). *STARD6* (ENSG00000174448) shows interactions in five tissue types, *C18orf54* (ENSG00000166845) also shows interactions in five types of tissue, and *CCDC68* (ENSG00000166510) shows interactions in four tissues. Each of these genes interacts with loci 49 and 50. Gene-based statistics derived in MAGMA indicated a role for 277 genes (Supplementary Table 13), 73 of which overlapped with genes implicated by all three mapping strategies (Fig. 3a).

For the anxiety/tension special factor, positional mapping indicated a role for 69 genes, with eQTL mapping indicating a role for 111 genes. Chromatin interaction mapping annotated a total of 136 genes (Supplementary Table 14 & 15, Supplementary Figure 2). Across these three mapping strategies, 222 unique genes were identified with 70 of these being implicated by two mapping strategies. A total of 24 unique genes were implicated by all three mapping strategies. MAGMA was also used to indicate a role for 99 genes (Supplementary Table 16). Fifteen of these genes overlapped with those identified using the three mapping strategies (Fig. 3b).

Sixteen genes were identified using positional mapping for the worry/vulnerability special factor. eQTL mapping matched cis-eQTL SNPs to 16 genes and 58 genes were identified using chromatin interactions between the SNPs’ genomic regions, and close or distant genes (Supplementary Table 17 & 18, Supplementary Figure 3**)**. For the worry/vulnerability special factor these mapping strategies implicated 71 unique genes of which 14 were implicated by two mapping strategies, and five were implicated by all three. Using MAGMA, 65 genes were implicated (Supplementary Table 19), five of which overlapped with genes implicated by all three mapping strategies (Fig. 3c).

### Cross-trait comparison of implicated genes

Next, we examined the genes implicated by each of the four methods across the three factors of neuroticism. This analysis revealed little overlap in the genes implicated as potentially being causal for each of the three factors. Using positional mapping, whilst 294 genes were implicated for the general factor of neuroticism and 69 were implicated for anxiety/tension only six genes were implicated across both neuroticism phenotypes. A total of 16 genes were implicated for the worry/vulnerability phenotype with only three genes overlapping with the 294 implicated for the general factor. No genes were shared between the anxiety/tension special factor and the worry/vulnerability special factor using positional mapping (Fig. 3d).

Using eQTL mapping, 329 genes were implicated for the general factor of neuroticism and 111 for the anxiety/tension special factor with 14 genes being found to be associated with both of these phenotypes. No other overlaps occurring using eQTL mapping (Fig. 3e). Chromatin interaction mapping indicated that 478 genes were implicated in the general factor of neuroticism and 136 were implicated for the anxiety/tension special factor, with 20 genes found to be shared between these two neuroticism phenotypes. A total of 58 genes were implicated for the worry/vulnerability special factor of neuroticism with 11 in common with the general factor (Fig. 3f).

Finally, using MAGMA, eight genes were found to be common in the 277 genes implicated for the general factor of neuroticism and the 99 implicated for the anxiety/tension special factor. A total of 65 genes were implicated for worry/vulnerability, 20 of which were also associated with the general factor of neuroticism and nine were found to share an association with the anxiety/tension special factor. No genes were found to be associated with all three phenotypes (Fig. 3g).

Of the 73 genes that were implicated as being linked to the general factor of neuroticism by positional mapping, eQTL mapping, and chromatin interaction mapping, 71 showed no overlap with either of the two special factors. These genes that showed a unique association with the general factor of neuroticism include *MAPT*, which encodes the microtubule-associated protein tau. *MAPT* transcripts are differentially expressed within the nervous system and mutations in *MAPT* have been linked to neurodegenerative disorders, such as Alzheimer’s disease, frontotemporal dementia, and cortico-basal degeneration.

A total of 13 genes showed evidence of being linked to the anxiety/tension special factor of neuroticism by the four gene prioritization methods without being associated with either the general factor of neuroticism or to the worry/vulnerability special factor. Of these genes, *DOC2A* was of note as it is mainly expressed in the brain and is involved in Ca(2+)-dependent neurotransmitter release. Also, uniquely associated with the anxiety/tension special factor was *BDNF* (brain-derived neurotrophic factor), which encodes a member of the nerve growth factor of proteins. The expression of this gene has been found to be reduced in those suffering from Alzheimer’s disease, Parkinson’s disease, and Huntington’s disease. *BDNF* has also been implicated in the regulation of the stress response as well as the biology of mood disorders. There were also five unique genetic associations with the worry/vulnerability special factor that were identified using the four methods of prioritising genes. *GRM8* (Glutamate metabotropic receptor 8) was one such gene and is linked to G-protein coupled receptor activity and group III metabotropic glutamate receptor activity. *GRM8* has been associated with schizophrenia.

### Gene-set and gene-property analysis

Gene-set analysis provided further evidence that special factors of anxiety/tension and worry/vulnerability had a different underlying biology compared with the general factor of neuroticism. Eight gene sets attained statistical significance for the general factor of neuroticism (Supplementary Table 20), two of which, neurogenesis (gene-set size = 13,56 genes, *P* = 6.16 × 10^−8^) and neuron spine (gene-set size = 116 genes, *P* = 2.95 × 10^−6^), have been found to be significantly enriched for neuroticism previously using the sum-score method to derive a neuroticism measure [15]. No gene sets were found to be enriched for the anxiety/tension special factor (Supplementary Table 21). Two gene sets were found to be significant for the worry/vulnerability special factor (Supplementary Table 22), high voltage gated calcium channel activity (gene-set size = 10 genes, *P* = 1.14 × 10^−6^), and voltage gated calcium channel complex (gene-set size = 39 genes, *P* = 4.03 × 10^−6^).

Using all 18,330 genes from the MAGMA analysis, a gene-property analysis was conducted. The gene-property analysis showed a significant relationship between the gene’s level of transcription in the brain and their level of association with each of the three factors (general factor *P* = 8.64 × 10^−7^, anxiety/tension special factor *P* = 9.06 × 10^−8^, worry/vulnerability special factor *P* = 1.06 × 10^−7^ (Supplementary Table 23-25). The direction of effect indicated that the greater the level of association a gene had with each of the three phenotypes, the greater its level of expression in the brain. This relationship can be seen to encompass multiple regions across the cortex (Supplementary Table 26-28). A gene’s level of expression within the brain in the early-mid prenatal period was predictive of its association with the general factor of neuroticism (*P* = 0.004) and the worry/vulnerability special factor (*P* = 0.001) (Supplementary Table 29–31). None of the age group specific transcription groupings were significant (Supplementary Table 32-34).

### Genetic correlations with cognitive, psychiatric, socioeconomic status (SES), health, anthropometric, and reproductive traits

**Fig. 4:**
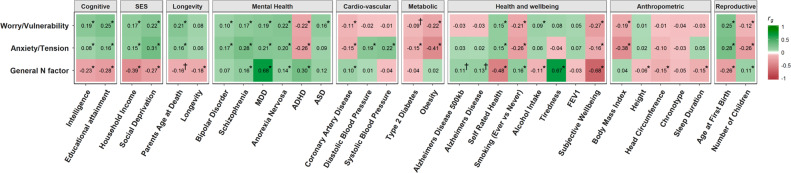
Genetic correlations between the general factor of neuroticism, the anxiety/tension special factor, the worry/vulnerability special factor with 31 cognitive/socioeconomic/health traits. Colour indicates the direction of the correlation and shade indicates the magnitude of the correlation. Asterisk indicates statistical significance after controlling for 32 tests using FDR, and a dagger indicates nominal significance that did not withstand FDR correction. Both the social deprivation and self-rated health phenotype had the scales reversed so that a greater score reflected a greater level of self-rated health and higher socioeconomic status [9, 75]. *ADHD* attention-deficit/hyperactivity disorder, *ASD* autism spectrum disorder, *MDD* major depressive disorder, *FEV1* forced expiratory volume in 1 second

Next, we examined the overlap between the genome-wide polygenic signal of the three neuroticism factors with health, anthropometric, SES, longevity, reproductive, and well-being variables. Following FDR correction for multiple comparisons [41], 20 of the 32 genetic correlations were statistically significant for the general factor of neuroticism, 21 of the 32 were significant for the anxiety/tension special factor, and 20 of the 32 were statistically significant for the worry/vulnerability special factor (Fig. 4 and Supplementary Table 35).

The general factor of neuroticism showed significant negative genetic correlations with the cognitive variables (intelligence, *r*_g_ = −0.23, *P* = 1.83 × 10^−23^, educational attainment, *r*_g_ = −0.28, *P* = 1.43 × 10^−47^). By contrast, the anxiety/tension and the worry/vulnerability special factor showed significant positive genetic correlations with intelligence (anxiety/tension, *r*_g_ = 0.08, *P* = 0.002, worry/vulnerability, *r*_g_ = 0.19, *P* = 4.12 × 10^−13^) and with educational attainment (anxiety/tension, *r*_g_ = 0.16, *P* = 6.86 ×10^−13^, worry/vulnerability, *r*_g_ = 0.25, *P* = 3.07 × 10^−25^).

Such contrasting findings were also evident when examining SES variables. The genetic variants associated with a higher level of the general factor of neuroticism were associated with a greater genetic risk for a lower household income (*r*_g_ = −0.39, *P* = 8.56 × 10^−24^) and living in an area with a higher level of social deprivation (*r*_g_ = −0.27, *P* = 5.18 × 10^−10^). On the other hand, the anxiety/tension and the worry/vulnerability special factors showed significant genetic correlations in the opposite directions to the general factor of neuroticism for household income (anxiety/tension *r*_g_ = 0.15, *P* = 0.0019, worry/vulnerability *r*_g_ = 0.17, *P* = 2.22 × 10^−4^) and living in an area with a lower level of social deprivation (anxiety/tension *r*_g_ = 0.31, *P* = 1.04 × 10^−8^, worry/vulnerability *r*_g_ = 0.22, *P* = 7.94 × 10^−5^). This indicates that, whereas neuroticism may be associated with lower SES, the two special factors measured here appear to be associated with advantages that, in turn, are associated with the acquisition of wealth, and improved living conditions.

This difference between the genetic correlations derived using the general factor and the worry/vulnerability and the anxiety/tension special factors was also found for reproductive traits. For age at first birth, the anxiety/tension and worry/vulnerability special factors were genetically correlated with delaying childbirth (anxiety/tension *r*_g_ = 0.28, *P* = 2.88 × 10^−16^, worry/vulnerability *r*_g_ = 0.25, *P* = 1.18 × 10^−13^) and having fewer children (anxiety/tension *r*_g_ = −0.26, *P* = 1.07 × 10^−8^, worry/vulnerability *r*_g_ = −0.12, *P* = 0.004). On the other hand, the negative genetic correlation found between the general factor of neuroticism and the age of first birth (*r*_g_ = −0.26, *P* = 4.37 × 10^−18^) and the positive genetic correlation found between the general factor and number of children (*r*_g_ = 0.11, *P* = 0.005) indicate that genetic variants that are associated with higher general neuroticism are also associated with a lower age at first birth and having more children.

Self-rated health and longevity also showed this pattern of results, whereby significant genetic correlations were identified for the three traits, but with the opposite direction of effect between the general factor and the two special factors. For the general factor, a negative genetic correlation was found with longevity (*r*_g_ = −0.18, *P* = 0.006), a nominally significant negative genetic correlation was found with parents’ age at death (*r*_g_ = −0.16, *P* = 0.049), and a negative genetic correlation was found with self-rated health (*r*_g_ = −0.48, *P* = 1.55 × 10^−22^). For the anxiety/tension and worry/vulnerability special factors, positive genetic correlations were found with parental longevity, (anxiety/tension, *r*_g_ = 0.16, *P* = 0.023; worry/vulnerability, *r*_g_ = 0.21, *P* = 0.002) and with self-rated health (anxiety/tension, *r*_g_ = 0.15, *P* = 3.92 × 10^−4^; worry/vulnerability, *r*_g_ = 0.15, *P* = 1.16 × 10^−4^). This pattern of genetic correlations indicates that the genetic variants associated with an increase in both special factors are also associated with better self-rated health, as well as longer life.

Importantly, there were also phenotypes where the genetic correlations with each of the three factors of neuroticism showed a consistent direction of effect. As one would expect, this was observed for mental health variables, including schizophrenia (general factor *r*_g_ = 0.16, *P* = 1.22 × 10^−8^, anxiety/tension *r*_g_ = 0.28, *P* = 7.66 × 10^−24^, worry/vulnerability *r*_g_ = 0.17, *P* = 4.77 × 10^−10^), major depressive disorder (general factor *r*_g_ = 0.68, *P* = 1.28 × 10^−17^, anxiety/tension *r*_g_ = 0.21, *P* = 0.003, worry/vulnerability *r*_g_ = 0.19, *P* = 0.01), and anorexia nervosa (general factor *r*_g_ = 0.14, *P* = 1.87 × 10^−6^, anxiety/tension *r*_g_ = 0.20, *P* = 9.63 × 10^−9^, worry/vulnerability *r*_g_ = 0.22, *P* = 1.05 × 10^−12^). A consistent direction of effect was also observed for the genetic correlations with subjective well-being (general factor *r*_g_ = −0.68, *P* = 2.75 × 10^−71^, anxiety/tension *r*_g_ = −0.15, *P* = 0.0004, worry/vulnerability *r*_g_ = −0.27, *P* = 1.36 × 10^−9^). This consistent direction of effect indicates that the directional changes seen with the other categories of traits are not a product of how the special factors were constructed, or a product of collider bias, as each of the three factors measured using the same neuroticism scale make independent contributions that are linked to worse mental health.

The two special factors also showed unique genetic correlations with other variables. In the case of anxiety/tension, a positive genetic correlation was found with both systolic (*r*_g_ = 0.22, *P* = 1.94^−6^) and diastolic blood pressure (*r*_g_ = 0.19, *P* = 2.46 × 10^−4^), indicating that the genetic burden for the anxiety/tension special factor is associated with unique health problems not associated with the genetic risk of high levels of worry/vulnerability.

### Genetic prediction

Polygenic risk score analysis was first used to replicate the overall polygenic signal associated with the general factor of neuroticism, and the two special factors into an independent sample. Polygenic risk scores were created using each of the three neuroticism phenotypes in UK Biobank in order to predict each of the three neuroticism phenotypes in GS:SFHS using five *P*-value thresholds. This led to 15 analyses per neuroticism phenotype (i.e., the general factor of neuroticism in UK Biobank was used to predict a general factor of neuroticism in GS:SFHS using five *P*-value cut offs, the anxiety/tension special factor in GS:SFHS using five *P*-value cut offs, and the worry/vulnerability special factor in GS:SFHS using five *P*-value cut offs). In each of these sets of 15 tests, the greatest amount of variance was explained when the factor of neuroticism that was used to construct the PGRS was the same as that being predicted. The PGRS for the general factor of neuroticism explained between 1.42 and 2.22% of the phenotypic variance of general neuroticism, but only explained between 0.16 and 0.22% of the anxiety/tension special factor and between 0.04 and 0.18% of the worry/vulnerability special factor (Supplementary Table 36).

This same pattern was seen when deriving polygenic risk scores using the special factors. The special factor of anxiety/tension predicted between 0.40 and 0.45% of the special factor of anxiety/tension, but only between 0.16 and 0.33% of the general factor of neuroticism and between 0.24 and 0.39% of the worry/vulnerability special factor. Finally, the worry/vulnerability special factor predicted between 0.67 and 0.97% of worry/vulnerability, but only between 0.15 and 0.44% of the general factor of neuroticism and only 0.09 and 0.12% of the special factor of anxiety/tension (Supplementary Table 36).

Using polygenic risk scores we were able to predict variance in a number of phenotypes that showed genetic correlations with each of the three factors of neuroticism. Predicting household income and intelligence in GS:SFHS we find that, when using the general factor of neuroticism to derive the PGRS, the beta weights from each of the five PGRS were negative and the PGRS with the greatest explanatory power for income used a *P*-value cut-off of 0.05 predicting 0.26% of variance in household income (Beta = −0.051, SE = 0.012, *P* = 3.16 × 10^−5^); for intelligence, a *P-*value cut-off of 1 explained the most variance at 0.24% (Beta = −0.049, SE = 0.012, *P* = 2.54 × 10^−5^). When constructing the PGRS using the two special factors, positive beta weights were observed when predicting both intelligence and income. For anxiety/tension, the PGRS with the greatest explanatory power when predicting household income used a *P*-value cut-off of 0.05 and explained 0.09% of the variance (Beta = 0.030, SE = 0.012, *P* = 0.015). When predicting intelligence using anxiety/tension a *P*-value cut-off of 0.01 explained 0.03% of intelligence (Beta = 0.016, SE = 0.012, *P* = 0.161). Although this was not significant, it is consistent with the genetic correlations presented as, out of the three neuroticism phenotypes, anxiety/tension has the smallest genetic correlation with intelligence.

Polygenic prediction using the special factor of worry/vulnerability showed that the *P*-value cut-off of 1 predicted the most variance, 0.06%, again with a positive beta (Beta = 0.024, SE = 0.012, *P* = 0.47). For intelligence, a *P*-value cut of 0.01 was the most predictive, explaining 0.12% where a positive beta was observed (Beta = 0.032, SE = 0.012, *P* = 0.006) (Supplementary Table 36).

## Discussion

Higher neuroticism has been shown to be associated with worse physical and mental health, as well as a shorter lifespan [6]. However, much research is based on measures in which individual items on neuroticism scales are summed [19] to generate a single overall score, a method that may occlude the effects of the heterogeneous traits that make up neuroticism. This study used a bifactor model to estimate scores relating to a general neuroticism factor and to two special factors of neuroticism. By quantifying the variance that is common across each of the 12 items, a general factor of neuroticism was identified and extracted. In addition to the extraction of this general factor, two special factors were identified, which we termed anxiety/tension and worry/vulnerability. These special factors can be seen to measure aspects of neuroticism that are independent from the general factor and may be conceptualized as the aspects of neuroticism that are uncorrelated with much of what is typically measured in a neuroticism battery; nevertheless, following the removal of variance that is common across all 12 items, they are still related to a subset of the neuroticism items. Anxiety/tension, therefore, is primarily composed of the residual variance from three items (“Would you call yourself a nervous person?”, “Would you call yourself tense or ‘highly strung’?”, “Do you suffer from ‘nerves’?”) that provide a measure of how nervous or tense a person is after the variance that is common across each of the 12 items is removed. As such, this special factor can be thought of as the degree to which an individual is anxious and tense given their overall level of neuroticism. Mutatis mutandis, the worry/vulnerability special factor can be interpreted in a similar manner. Both of these special factors were independent of the general factor.

Previous attempts to examine the heterogeneity of neuroticism using the UK Biobank data have used the sum-score approach, following the identification of items that are more strongly genetically correlated with each other [19]. However, although sub-factor scores derived in this way will contain variance specific to the sub-factor, they will also be contaminated with, depending on the items summed, a considerable amount of variance attributable to the general factor of neuroticism. This known issue [60] can seen in the work of Nagel et al. [19] who found that the genetic correlations between the depressed affect and worry sub-factors, with a general neuroticism factor were 0.86 and 0.84, respectively. Clearly, then, little can emerge from the ‘specific’ item-clusters of Nagel et al. [19] that is not, in fact, relevant to general neuroticism.

The sub-factor of worry in Nagel et al. [19] is a sum-score of the neuroticism items of “Would you call yourself a nervous person?”, “Are you a worrier?”, “Would you call yourself tense or ‘highly strung’?”, and “Do you suffer from ‘nerves’?”. In the current study we found that, independent of the variance that they shared with all 12 items, i.e., the general factor variance, the first, third, and fourth of these items loaded most strongly onto the anxiety/tension special factor identified by Gale et al. [6]. By comparing Fig. 3 of Nagel et al. [19] with Fig. 4 of the current study, one can see the effect that contamination from a general factor of neuroticism has. For example, Nagel et al. [19] found negative genetic correlations with intelligence (which they referred to as IQ) and with education, using their sum-score phenotype/item-cluster of “worry”; in the current study, however, the variation unique to being anxious and tense has a positive genetic correlation with both of these phenotypes. We also show a protective effect for genes that underlie individual differences in this factor, as they are associated with longer life and a reduced risk of coronary artery disease, in contrast to the findings of Nagel et al. [19].

Genome-wide association studies conducted on the general factor of neuroticism and the two special factors indicate that these are genetically heterogeneous traits at the SNP, gene, and biological pathway level. In addition, genetic correlations indicate that these phenotypes, derived using the same neuroticism scale, have distinct genetic etiologies that show a different overlap with health, cognitive, SES, reproductive, and anthropometric traits.

For the general factor of neuroticism, 51 independent genomic loci were identified, consistent with the original analysis of the UK Biobank data by Luciano et al. [15] and the re-analysis of the Luciano et al. [15] data sets by Nagel et al. [17], both of which used a sum-score measure of neuroticism. For the two special factors, fewer loci were identified, with 14 being associated with anxiety/tension and 10 for worry/vulnerability. Although there was very little overlap in these loci, functional annotation for each of these phenotypes produced highly similar results where independent genomic loci contained SNPs that were predominantly found in introns, and intergenic regions. This is in line with findings from GWAS conducted on other traits [9, 61] where the SNPs with the greatest effect were found in regions of the genome that are involved in gene expression, rather than in protein coding regions.

More similarity in the genetic etiology of these three neuroticism traits was seen when examining the relationship between gene expression in particular tissues and the gene-based test association statistics from MAGMA. In the current study, a gene’s level of expression in the brain was predictive of its association with each of the three phenotypes, a relationship that was found across the cortex. This indicates that common genetic variation in genes that are predominantly expressed in the brain are associated with each of the three neuroticism phenotypes.

Despite the similarity of the functional annotation of each of the three neuroticism phenotypes, there was a high level of divergence regarding which genes may be causal in these associations. As can be seen in Fig. 3a-c (and in Supplementary Figures 1-3), 104 genes were identified as possible causal genes underlying the associations found for the general factor using genomic position, eQTL information, and chromatin interaction data. Using the same methods, 24 genes were identified as potentially harbouring causal variation for the anxiety/tension special factor, and five genes for the worry/vulnerability special factor. The genes that were implicated for each of the three phenotypes showed very little overlap (Fig. 3d–f). This lack of overlap is also seen when examining the gene-based statistics, which also show that while a large number of genes were implicated, only a small number were implicated across two neuroticism phenotypes, with no genes being implicated across all three.

This lack of overlap in the genes implicated as being causally linked to the general factor of neuroticism and both the anxiety/tension and worry/vulnerability special factors indicates a divergent biology across the three neuroticism phenotypes. However, on examination of the genes that are unique to each of the three neuroticism phenotypes, it is clear that many have been linked to the activity and/or structure of the brain or are implicated in pathology that effects cortical tissues. For the anxiety/tension special factor, this included *DOC2A* and *BDNF*, which were both implicated using positional mapping, eQTL analysis, chromatin analysis, and gene-based statistics derived using MAGMA. *DOC2A* is mainly expressed in the brain and thought to be linked to Ca(2+)-dependent neurotransmitter release and has been previously linked to schizophrenia [62], a psychiatric disorder, in part, characterized by disorded cognitive functioning. Also linked to the anxiety/tension special factor was *BDNF*, which is also predominantly expressed in the brain and encodes a nerve growth factor and the binding of this protein to its receptor and promotes neuronal survival. Expression of *BDNF* has also been found to be reduced in patients with Alzheimer’s disease, Parkinson’s disease, and Huntington’s disease indicating its importance the healthy adult brain.

Although, for the worry/vulnerability special factor, only five genes were uniquely implicated using all four gene-finding strategies, the *GRM8* gene was among them. *GRM8*, like *DOC2A*, has been linked to schizophrenia in addition to sporadic Creutzfeldt–Jakob disease risk [63]. The *GRM8* gene codes for the protein mGluR8 that belongs to the metabotropic glutamate receptor family. This family has been associated with the transduction of physiological and cytotoxic signals mediated by the prion protein (PrP^C^). Other members of the metabotropic glutamate receptor family, mGluR1 and mGluR5, have been shown to interact with PrP^C^ and these associations appear to facilitate neurite outgrowth [64, 65]. Furthermore, it has been demonstrated that mGluR5 coupled with PrP^C^ mediates the cellular toxicity of soluble β-amyloid oligomers [65].

More biological differences were seen between the three neuroticism phenotypes when looking at the results of the gene-set analysis. For the general factor, eight gene sets were statistically significant once controlling for multiple tests. Of these eight, none were nominally significant for the anxiety/tension special factor, and, although four were nominally significant for the worry/vulnerability special factor, this did not withstand correction for multiple tests. The gene-set of neurogenesis was the most significant gene-set that was uniquely associated with the general factor of neuroticism. This result is consistent with what was found using a sum-score approach [14].

Neurogenesis is the process by which new neurons are created and this processes has previously been linked to intelligence in humans using GWAS data [9], and has been linked to cognitive flexibility in rodent models of cognitive ability [66–72]. This shared association between neurogenesis with both neuroticism and intelligence is unique to the general factor of neuroticism and, may serve to provide a partial explanation of the genetic correlation between intelligence, neuroticism, and mental health [73].

Two gene sets were also statistically significant for the worry/vulnerability special factor (high voltage gated calcium channel activity and the voltage gated calcium channel complex). These were unique associations with no nominal level of association being found with either the general factor or with the anxiety/tension special factor. Voltage gated calcium channels act as transducers of cell surface membrane potential changes into transient intracellular calcium that serves to initiate many kinds of physiological events. This temporary influx of calcium in response to membrane depolarisation serves to regulate many intracellular processes including neurotransmission and gene expression [74].

The results of the GWAS, its functional annotation, as well as the gene-based statistics, the gene-set and gene-property analyses, indicate that these neuroticism phenotypes are linked together regarding the greatest signal being found in regions of the genome involved in gene expression, and the total underlying polygenic signal clustering in genes that are predominately expressed in and across the brain. However, these phenotypes diverge when considering which genes are linked to the independent genomic loci, the genes that are mapped to them and which biological systems within the brain are implicated.

The pattern of genetic correlations seen with other phenotypes supports the findings that neuroticism, as derived using a sum-score method, is composed of multiple genetically heterogeneous phenotypes that have different genetic relationships to health, disease, and SES. This can most clearly be seen when examining the genetic correlations derived using cognitive, SES, longevity, and reproductive traits. As seen in Fig. 4, the general factor of neuroticism shows sizable negative genetic correlations with cognitive, SES, longevity, and reproductive traits (with the exception of number of children that displays a positive genetic correlation), whereas worry/vulnerability and anxiety/tension both show positive genetic correlations (with the exception of number of children that displays a negative genetic correlation). This indicates that the genetic risk for higher levels of the general factor of neuroticism is associated with an increased genetic risk of lower intelligence, lower levels of education, a lower level of SES, an earlier death, and more children being born at earlier ages, whereas the genetic variants linked with worry/vulnerability and anxiety/tension are linked to the genetic variants that facilitate intelligence, education, SES, are linked to a longer life, with fewer children being born, and giving birth at an older age.

Other traits from outside these categories display this same pattern, whereby the genetic risk for coronary artery disease is linked to the genetic risk for higher levels of general neuroticism but lower levels of anxiety/tension and worry/vulnerability. This reversal of the direction of effect is also seen when comparing genetic correlations derived from the general factor of neuroticism to those derived using the two special factors for self-rated health, smoking, and ADHD.

The general factor of neuroticism showed the strongest genetic correlations with major depressive disorder and subjective well-being. The magnitude of these genetic correlations (≥0.65), indicates a substantial genetic overlap. Of note is that the two special factors of neuroticism both display genetic correlations with major depressive disorder and subjective well-being that are in the same direction as those found using the general factor of neuroticism. This finding suggests that, in addition to a general factor of neuroticism, there are two sources of structured variance, indicating the presence of genetically heterogeneous phenotypes, also measured using a neuroticism scale. All three of these neuroticism phenotypes contribute independently to major depressive disorder, and to subjective well-being.

A consistent direction of effect was also observed for schizophrenia with positive genetic correlations identified for each of the three neuroticism phenotypes. In contrast to the genetic correlations with MDD and subjective well-being, the genetic correlation of the greatest magnitude was not with the general factor of neuroticism, but with the special factor of anxiety/tension (Fig. 4.). This indicates that much of the genetic link between schizophrenia and neuroticism is separate from what is common across the items used to measure neuroticism, and is best captured, not only by a subset of items, but also by a subset of the variance remaining once the variance that is common across all 12 items is removed.

Using polygenic risk scores we were able to show that the polygenic signal associated with the general factor of neuroticism, and each of the special factors, replicated into an independent sample. This can be seen as polygenic risk scores predicting the general factor of neuroticism in GS:SFHS, had the greatest explanatory power when derived using the general factor of neuroticism in UK Biobank. This pattern was also found for the two special factors where the special factor of anxiety/tension explained the most variance for the special factor of anxiety/tension, and the special factor of worry/vulnerability explained the most variance for the special factor of worry/vulnerability. This shows that the polygenic signal associated with each of the special factors is a better match to the same special factor derived an independent cohort, and not, as would be predicted if our factors were contaminated by variance associated with the general factor [19], simply another measure of general neuroticism.

The protective aspects of the polygenic signal associated with each of the special factors of anxiety/tension and worry/vulnerability was also replicated into GS:SFHS. This replication can be seen in that the polygenic burden for the general factor of neuroticism is predictive of lower SES, as measured using household income, and lower intelligence, whereas the polygenic burden for each of the special factors is associated with an increase in SES and intelligence.

Neuroticism is one of the most well-studied personality traits in the psychology and health literature, and it has been recognized as an important risk factor for personal, societal and financial woes in human societies [7]. The present work develops findings of neuroticism at the level of the phenotype [6, 35] by showing that neuroticism is ‘molecular’ and not ‘atomic’ at the genetic level also, with differently-valenced ‘atoms’ on these ‘molecules’ being related to both better and poorer human functioning.

## Supplementary information

Supplementary Tables 1 to 36

Supplementary File 1

Supplementary File 2

Supplementary Figure text

Supplementary Figure 1A CHR1

Supplementary Figure 1B CHR2

Supplementary Figure 1C CHR3

Supplementary Figure 1D CHR4

Supplementary Figure 1E CHR5

Supplementary Figure 1F CHR7

Supplementary Figure 1G CHR8

Supplementary Figure 1H CHR9

Supplementary Figure 1I CHR10

Supplementary Figure 1J CHR11

Supplementary Figure 1K CHR12

Supplementary Figure 1L CHR13

Supplementary Figure 1M CHR14

Supplementary Figure 1N CHR15

Supplementary Figure 1O CHR16

Supplementary Figure 1P CHR17

Supplementary Figure 1Q CHR18

Supplementary Figure 1R CHR22

Supplementary Figure 2A CHR1

Supplementary Figure 2B CHR2

Supplementary Figure 2C CHR3

Supplementary Figure 2D CHR4

Supplementary Figure 2E CHR6

Supplementary Figure 2F CHR8

Supplementary Figure 2G CHR10

Supplementary Figure 2H CHR11

Supplementary Figure 2I CHR12

Supplementary Figure 2J CHR16

Supplementary Figure 2K CHR18

Supplementary Figure 3A CHR2

Supplementary Figure 3B CHR4

Supplementary Figure 3C CHR5

Supplementary Figure 3D CHR7

Supplementary Figure 3E CHR11

Supplementary Figure 3F CHR13

Supplementary Figure 3G CHR18

Supplementary Figure 3H CHR21
