# Supplementary material for: Genetic contributions to two special factors of neuroticism are associated with affluence, higher intelligence, better health, and longer life
Source: Mol Psychiatry. 2019 Mar 13;25(11):3034–52. doi: 10.1038/s41380-019-0387-3 (PMC7577854; doi:10.1038/s41380-019-0387-3)
Supplement: Supplementary file 4 — Supplementary Figure text [file 41380_2019_387_MOESM4_ESM.docx]

Supplementary Figure 1-3.

Circos plots by chromosome illustrating genome-wide significant loci associated with the general factor of neuroticism (**Supplementary Figure 1** showing chromosomes 1, 2, 3, 4, 5, 7, 8, 9, 10, 11, 12, 13, 14, 15, 16, 17, 18, 22), the anxiety/tension special factor (**Supplementary Figure 2** showing chromosomes 1, 2, 3, 4, 6, 8, 10, 11, 12, 16, 18), and the worry/vulnerability special factor (**Supplementary Figure 3** showing chromosomes 2, 4, 5, 7, 11, 13, 18, 21) are shown. For each phenotype the most outer layer shows the Manhattan plot and only SNPs where *P* < 0.05 are shown. Each of the SNPs in the genomic risk loci are colour coded indicating the maximum r^2^ with one of the independent significant SNPs in the locus with red indicating the highest r^2^ and blue the lowest r^2^ (red r^2^ > 0.8, orange r^2^ > 0.6, green r^2^ > 0.4, and blue r^2^ > 0.2). SNPs shown in grey are not in LD with any of the genome wide significant SNPs. The rsID of the most significant lead SNP in each loci is shown. The second layer is the chromosomal ring with the independent genomic risk loci highlighted in blue. Next, the genes mapped by chromatin interactions or eQTLs are displayed. Genes mapped using chromatin interactions are displayed in orange, with genes mapped by eQTL shown in green. Genes that are displayed in red are those mapped using both chromatin interactions and eQTLs. Chromatin interaction links (coloured orange for chromatin interactions and green for eQTLs are displayed.
