# Supplementary figures and images for: Genetic contributions to two special factors of neuroticism are associated with affluence, higher intelligence, better health, and longer life
Source: Mol Psychiatry. 2019 Mar 13;25(11):3034–52. doi: 10.1038/s41380-019-0387-3 (PMC7577854; doi:10.1038/s41380-019-0387-3)

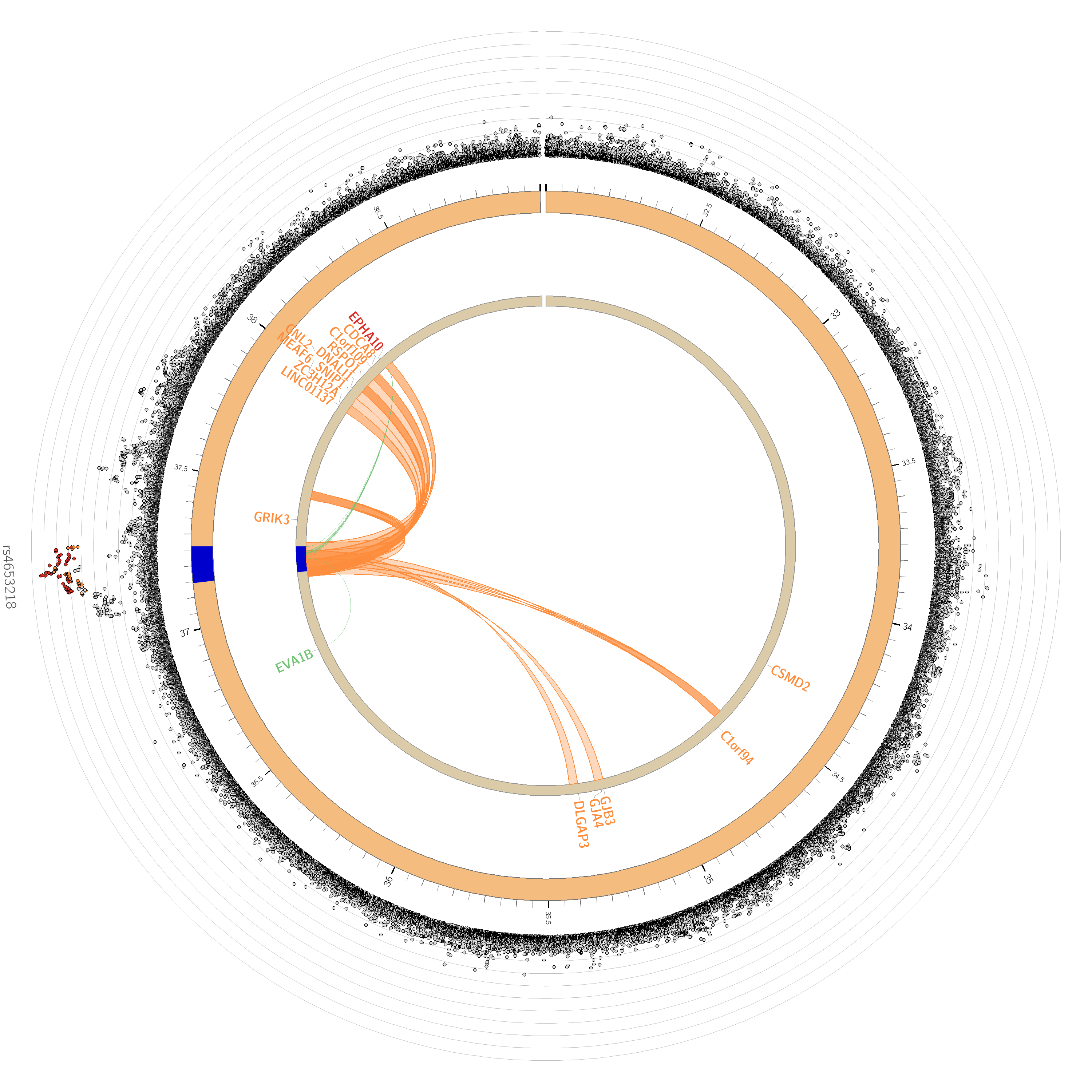

Supplement: Supplementary file 5 — Supplementary Figure 1A CHR1 [file 41380_2019_387_MOESM5_ESM.png]

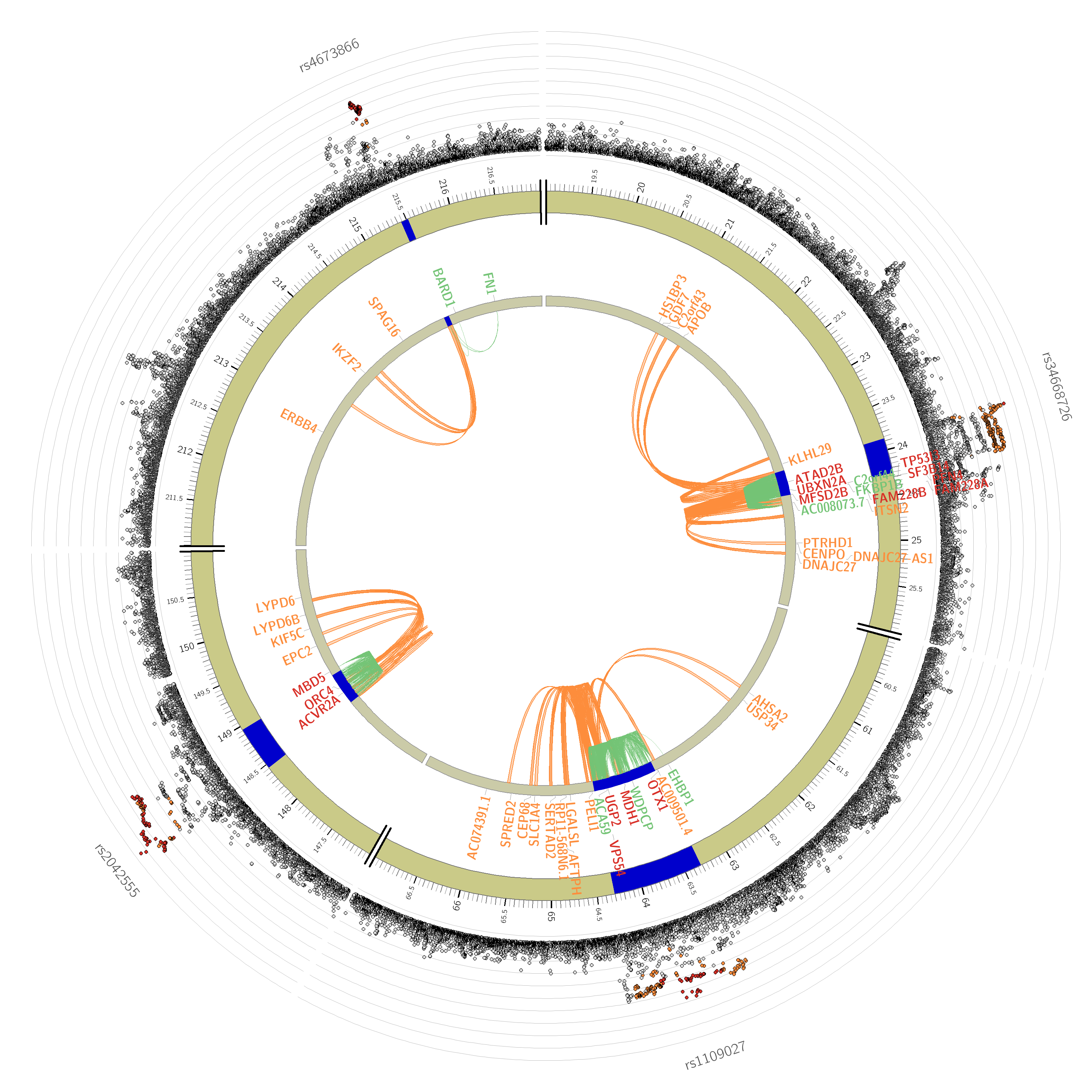

Supplement: Supplementary file 6 — Supplementary Figure 1B CHR2 [file 41380_2019_387_MOESM6_ESM.png]

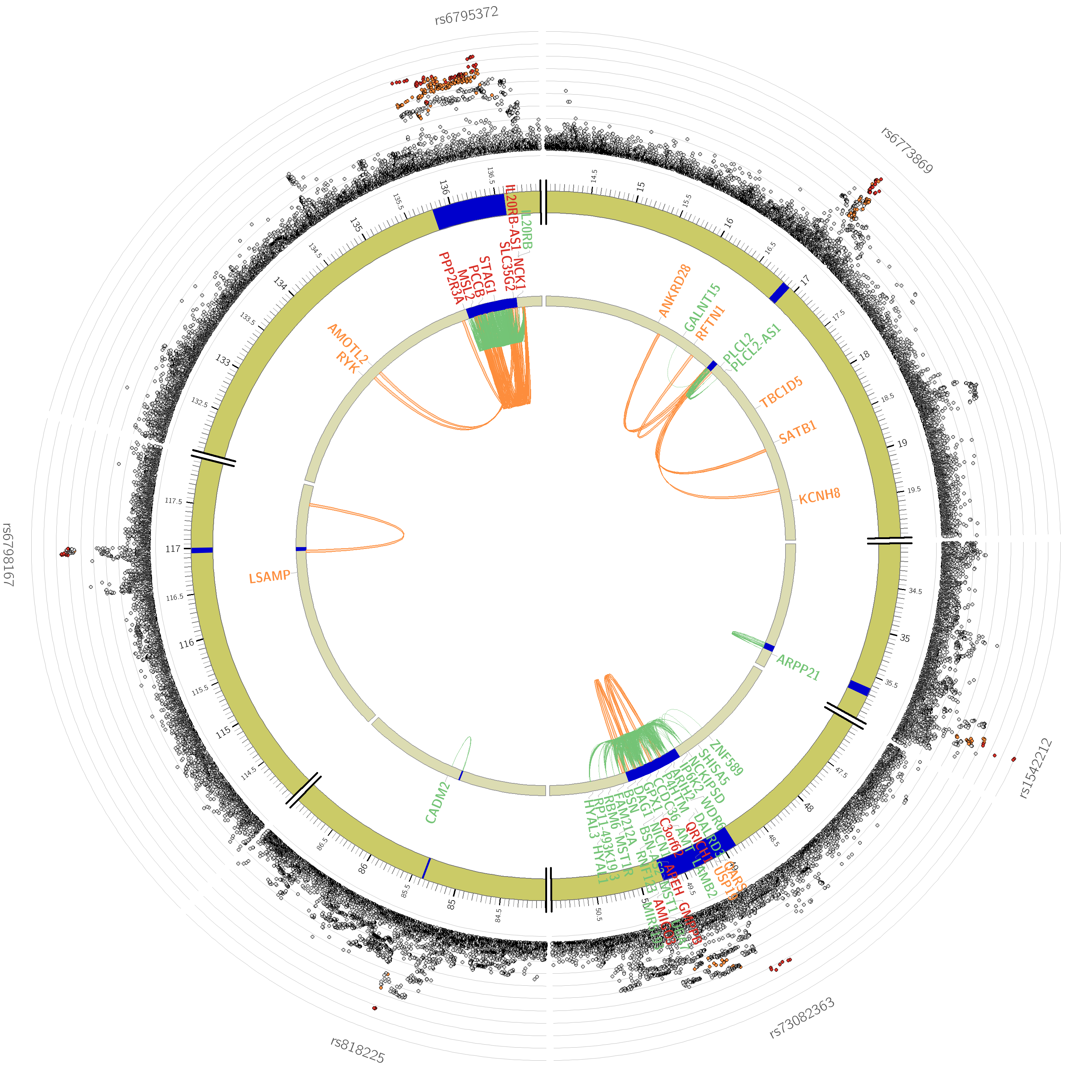

Supplement: Supplementary file 7 — Supplementary Figure 1C CHR3 [file 41380_2019_387_MOESM7_ESM.png]

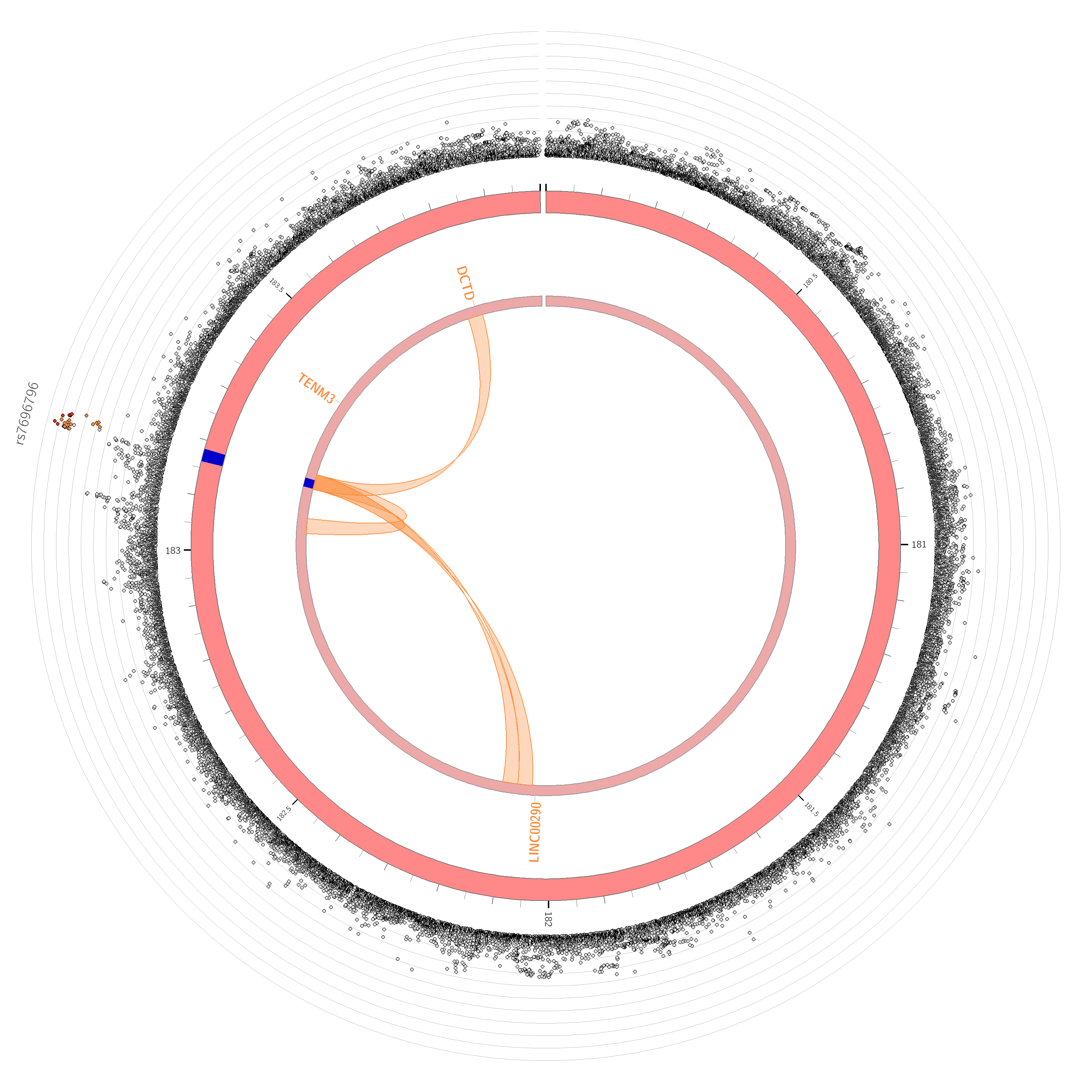

Supplement: Supplementary file 8 — Supplementary Figure 1D CHR4 [file 41380_2019_387_MOESM8_ESM.png]

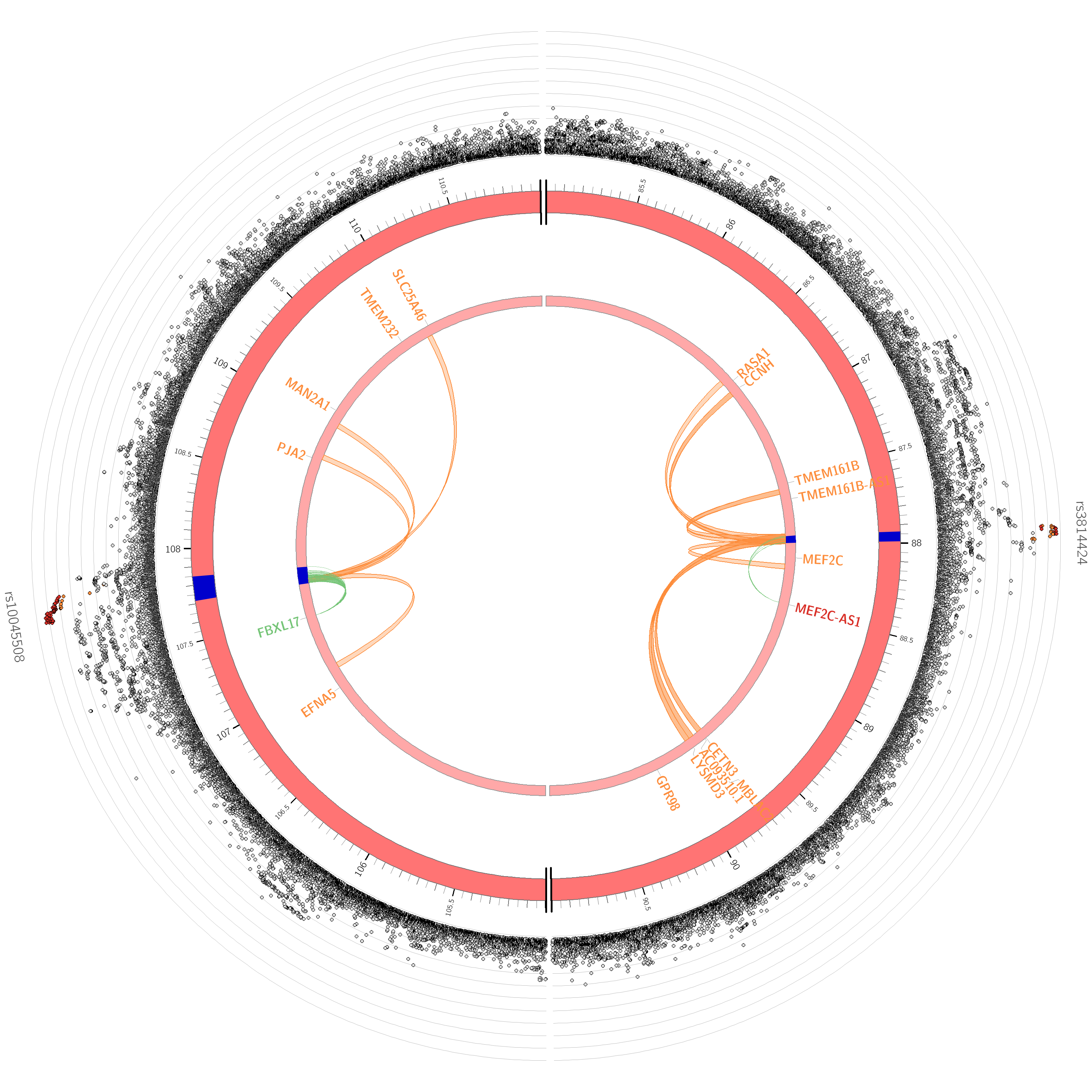

Supplement: Supplementary file 9 — Supplementary Figure 1E CHR5 [file 41380_2019_387_MOESM9_ESM.png]

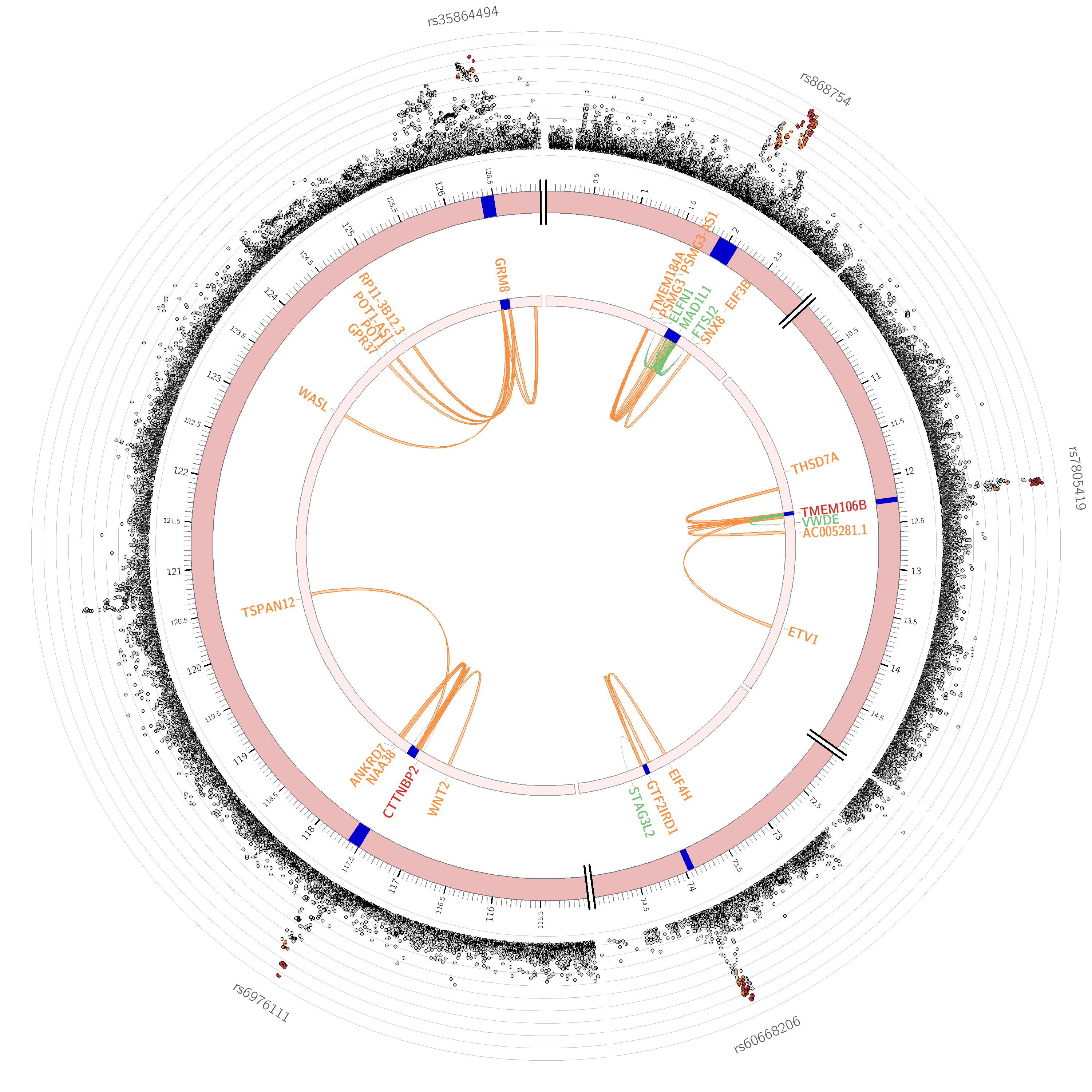

Supplement: Supplementary file 10 — Supplementary Figure 1F CHR7 [file 41380_2019_387_MOESM10_ESM.png]

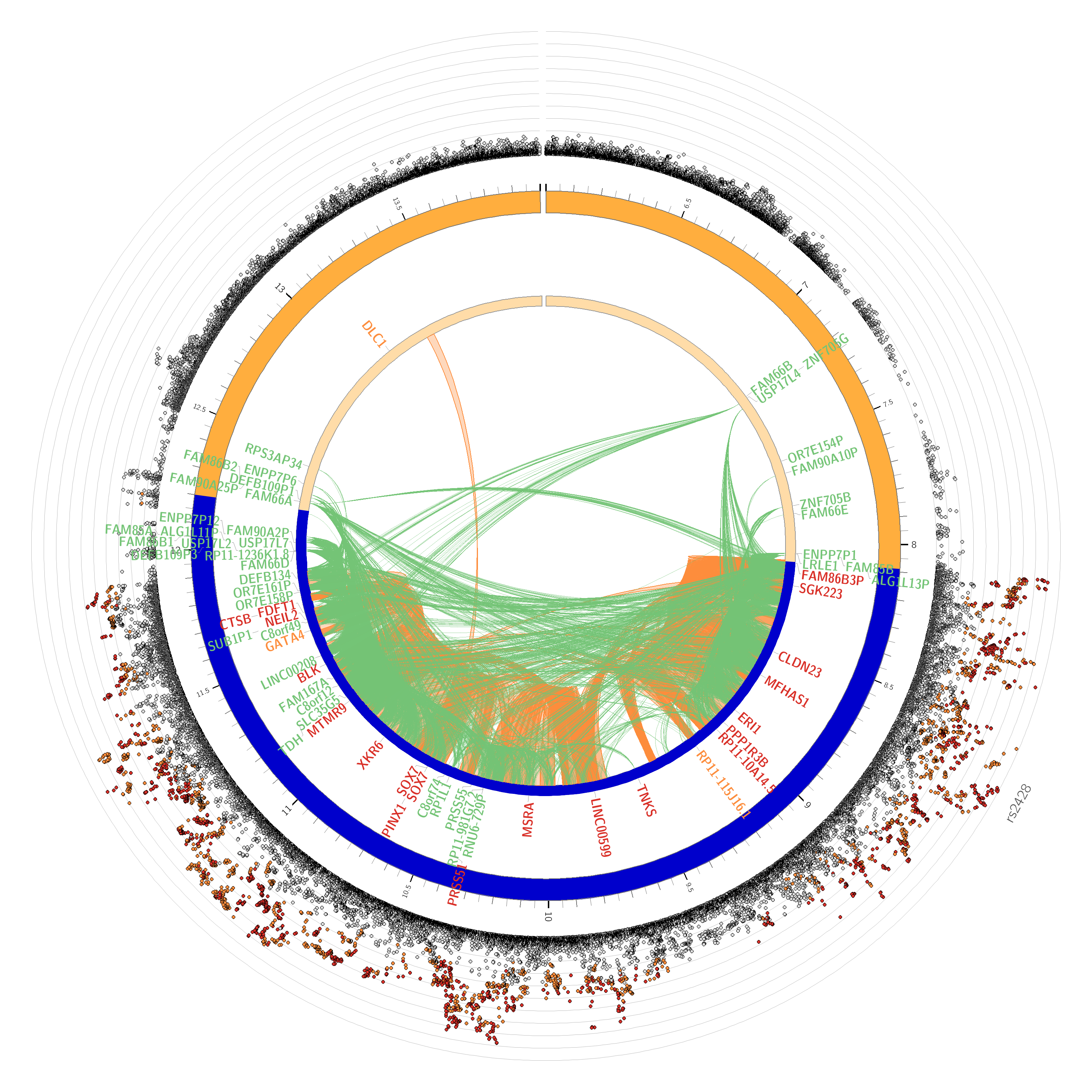

Supplement: Supplementary file 11 — Supplementary Figure 1G CHR8 [file 41380_2019_387_MOESM11_ESM.png]

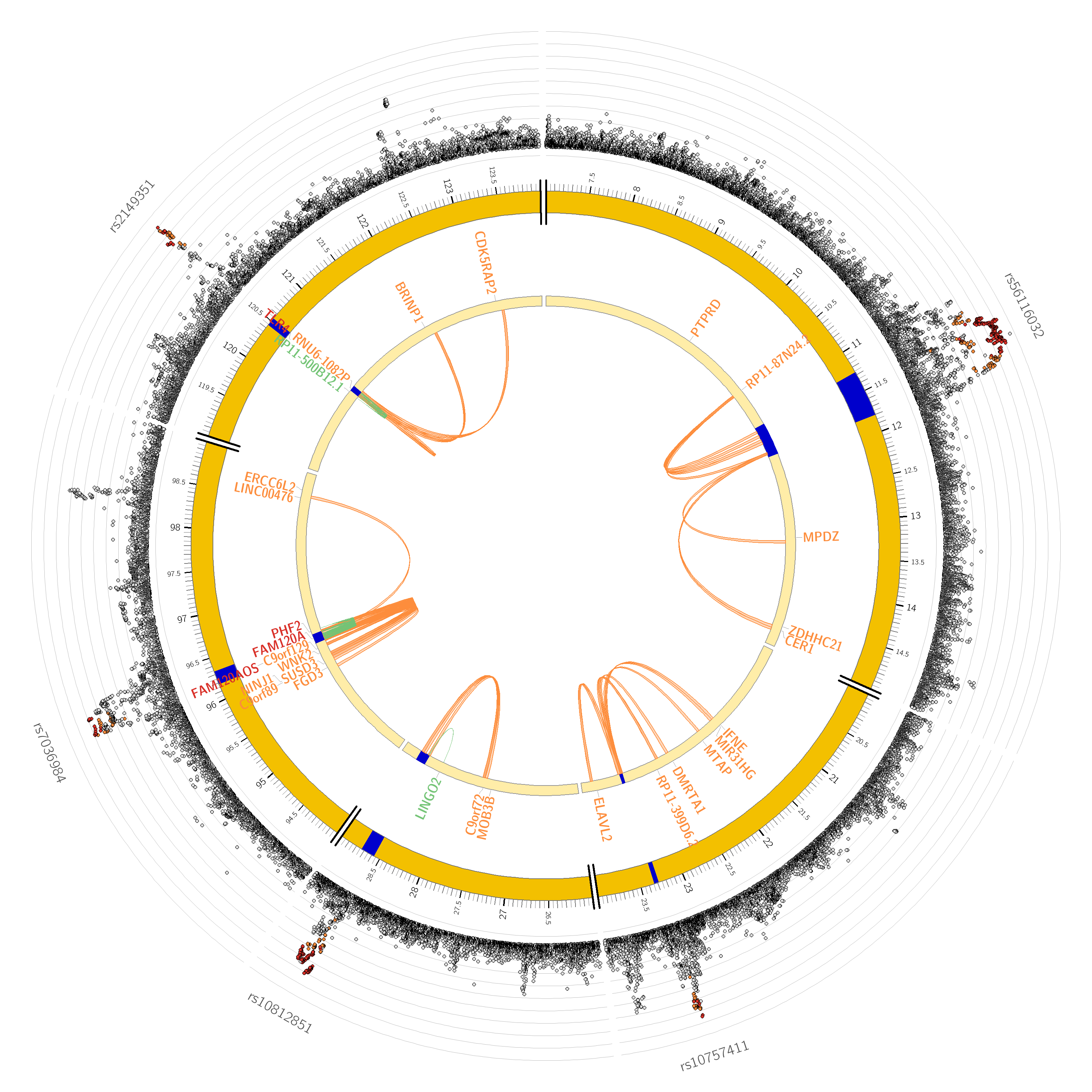

Supplement: Supplementary file 12 — Supplementary Figure 1H CHR9 [file 41380_2019_387_MOESM12_ESM.png]

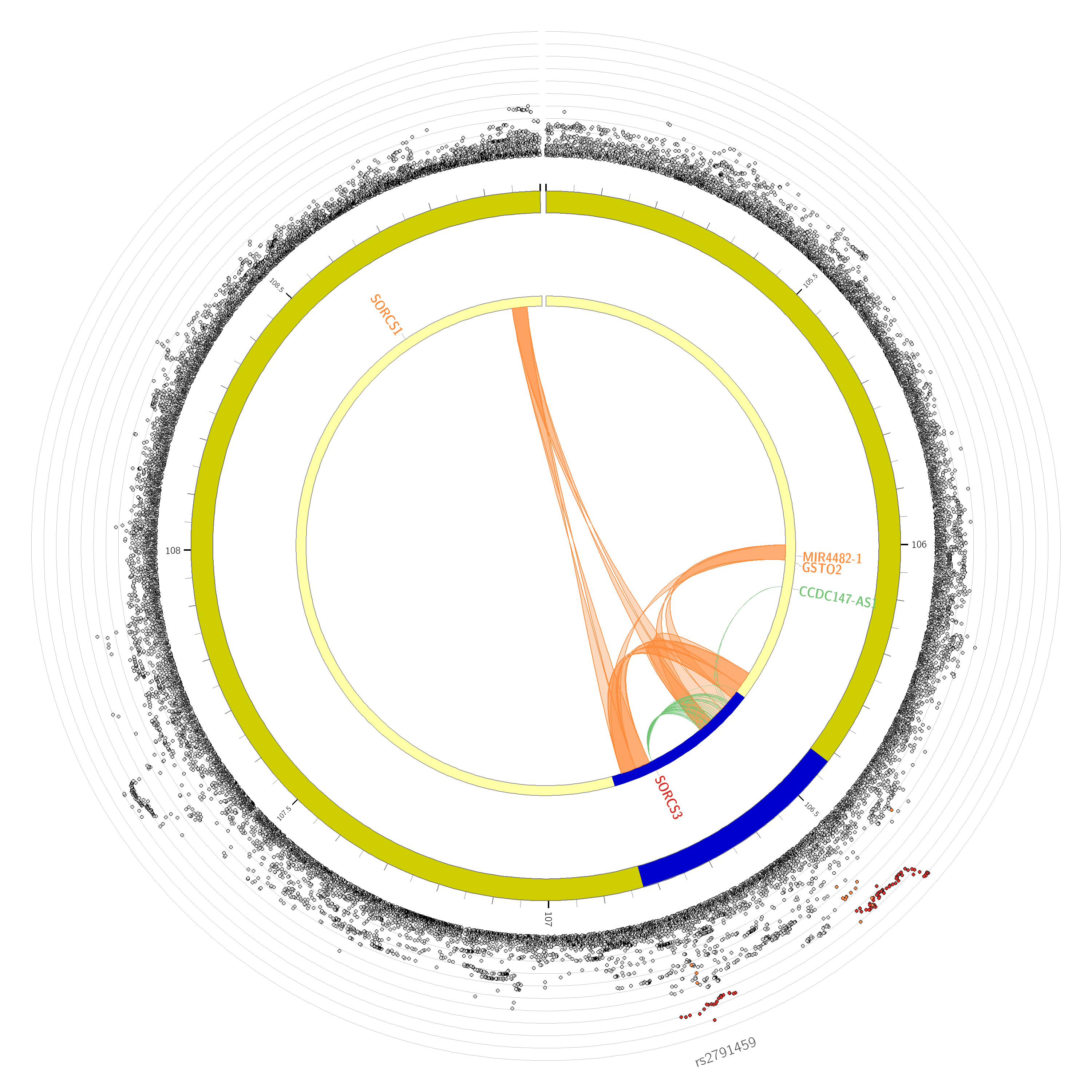

Supplement: Supplementary file 13 — Supplementary Figure 1I CHR10 [file 41380_2019_387_MOESM13_ESM.png]

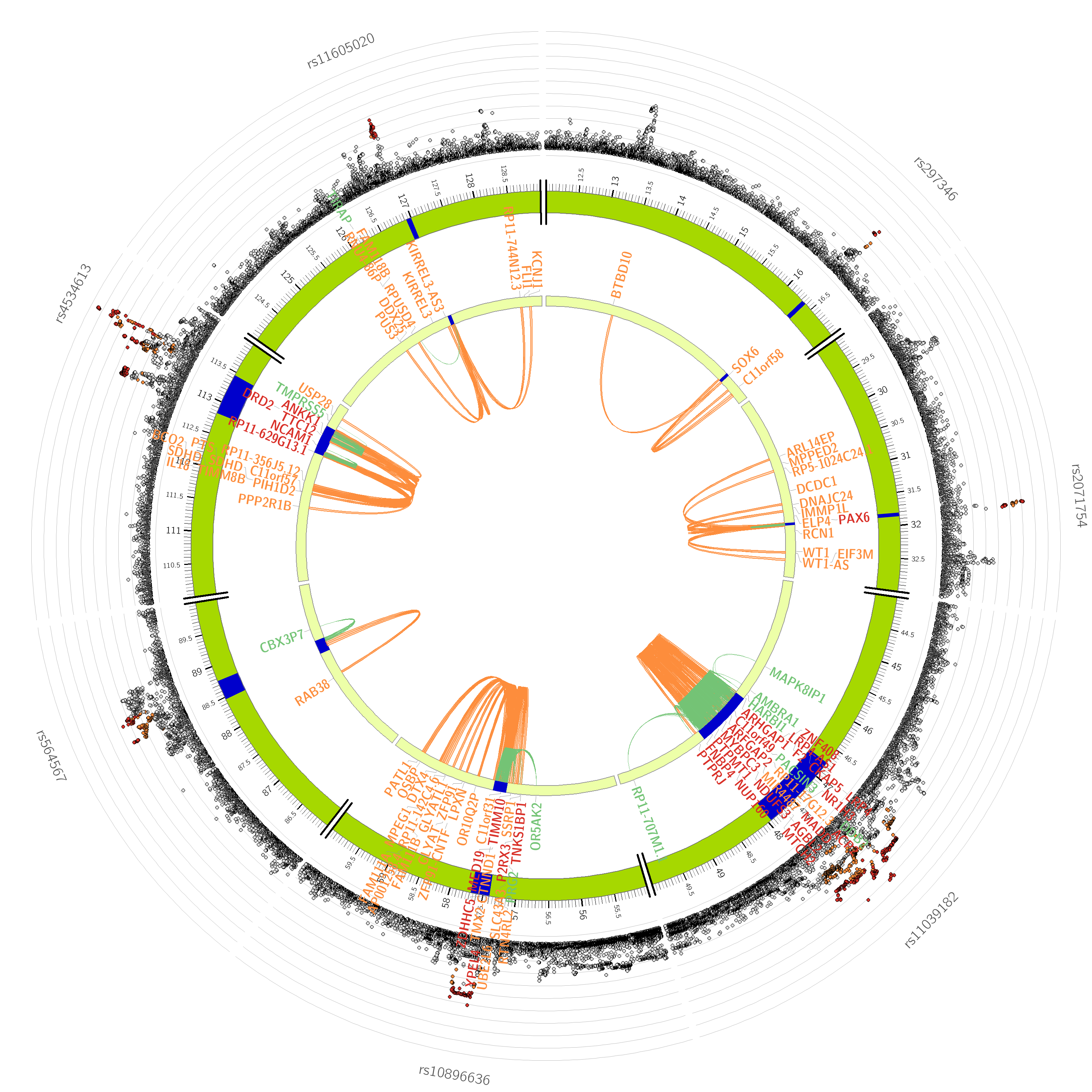

Supplement: Supplementary file 14 — Supplementary Figure 1J CHR11 [file 41380_2019_387_MOESM14_ESM.png]

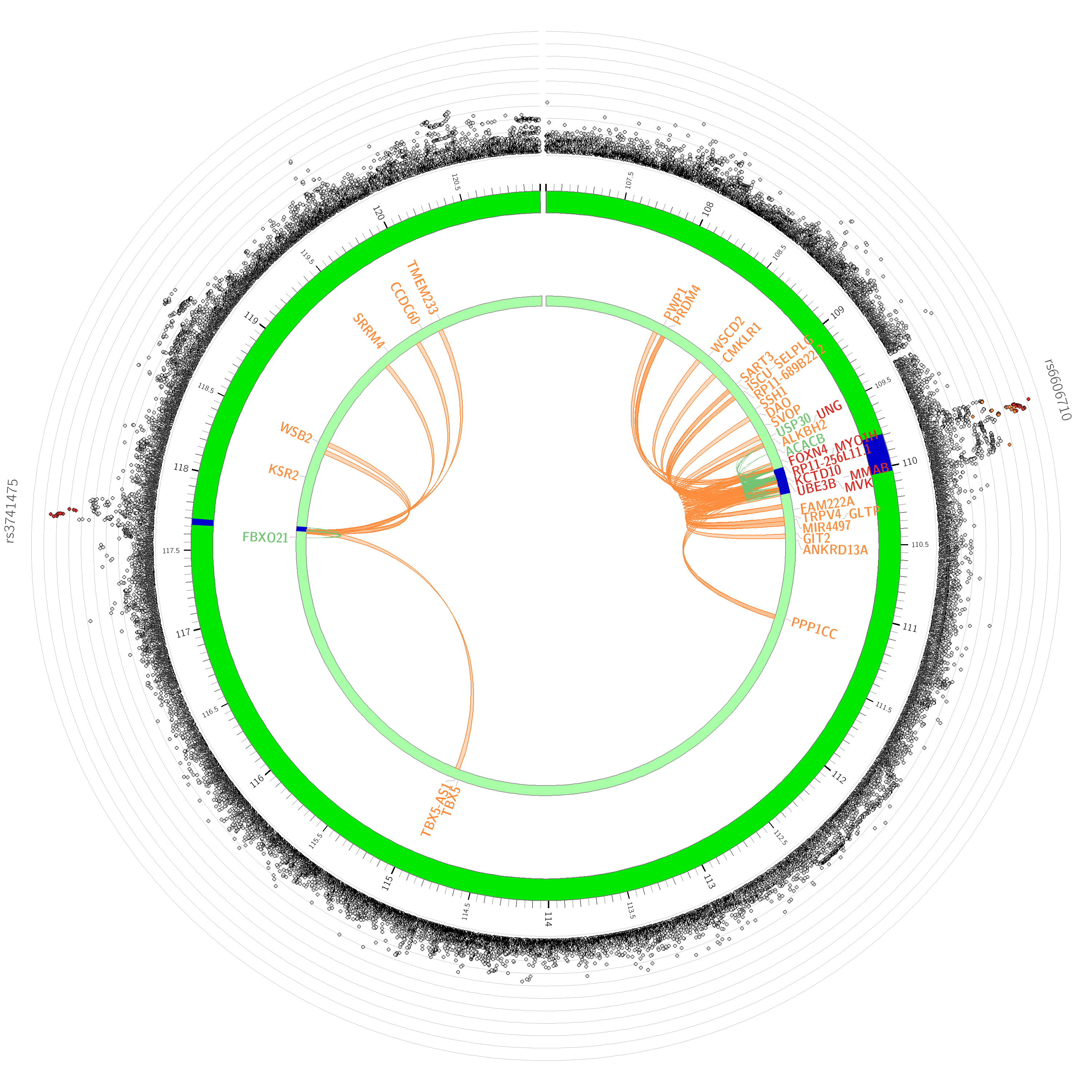

Supplement: Supplementary file 15 — Supplementary Figure 1K CHR12 [file 41380_2019_387_MOESM15_ESM.png]

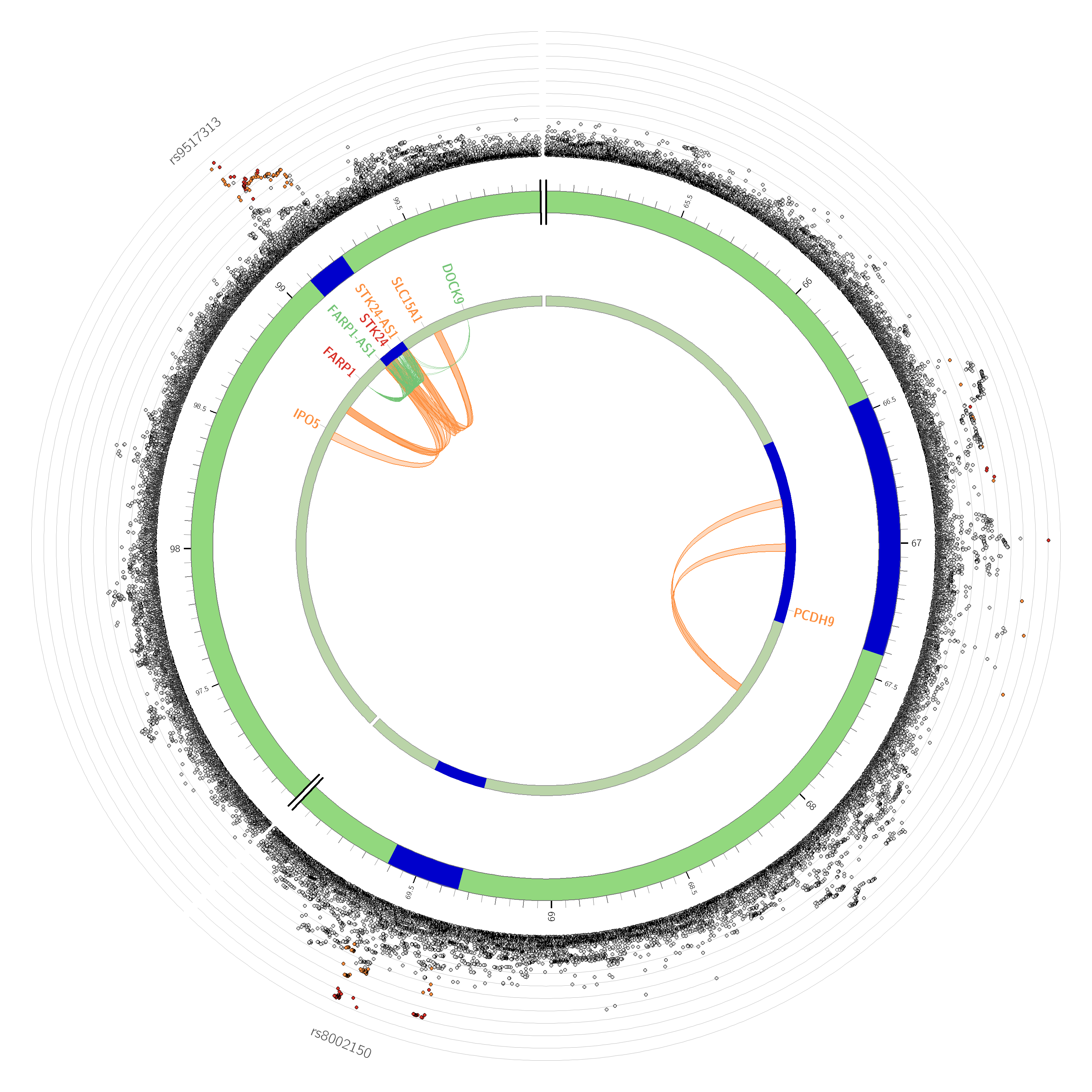

Supplement: Supplementary file 16 — Supplementary Figure 1L CHR13 [file 41380_2019_387_MOESM16_ESM.png]

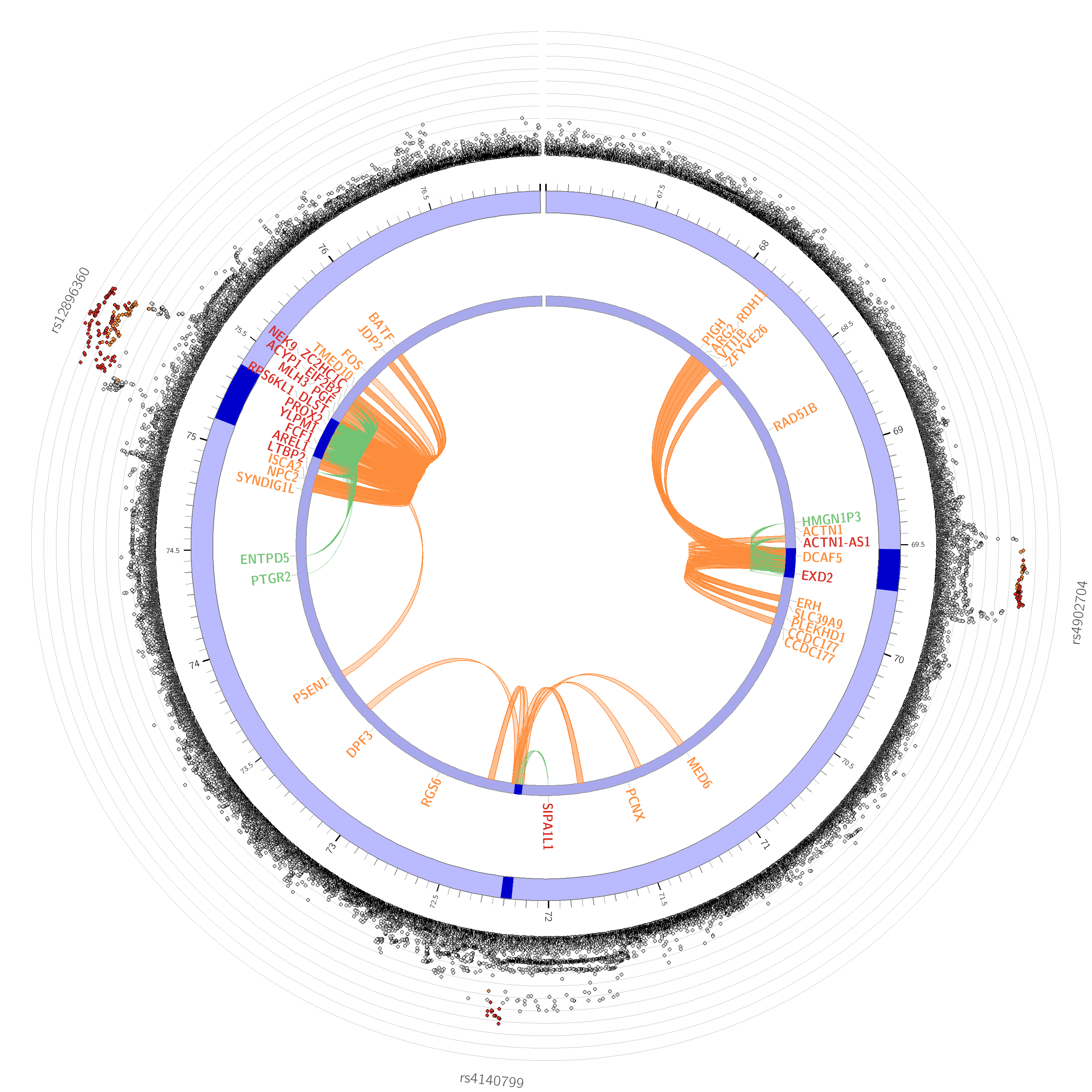

Supplement: Supplementary file 17 — Supplementary Figure 1M CHR14 [file 41380_2019_387_MOESM17_ESM.png]

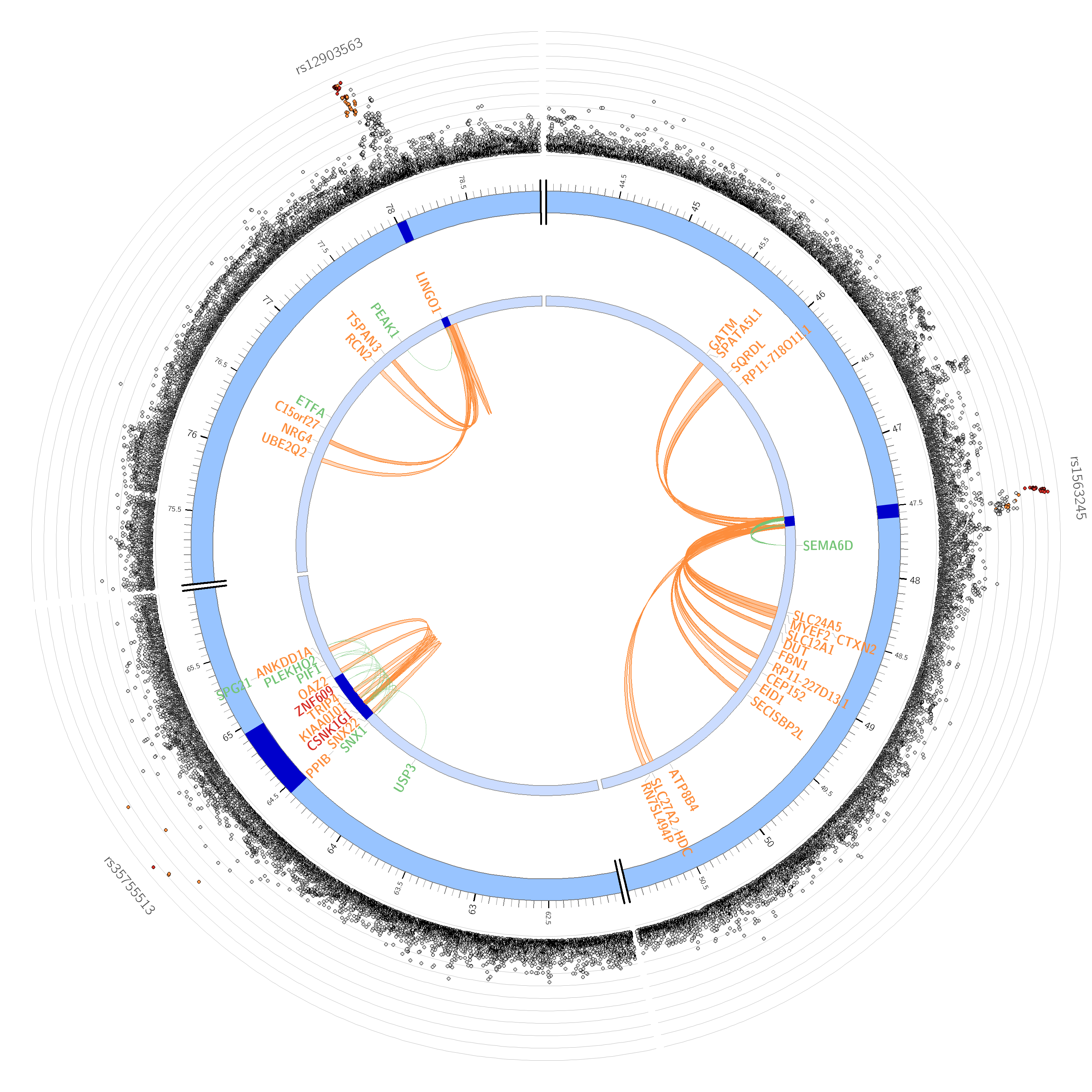

Supplement: Supplementary file 18 — Supplementary Figure 1N CHR15 [file 41380_2019_387_MOESM18_ESM.png]

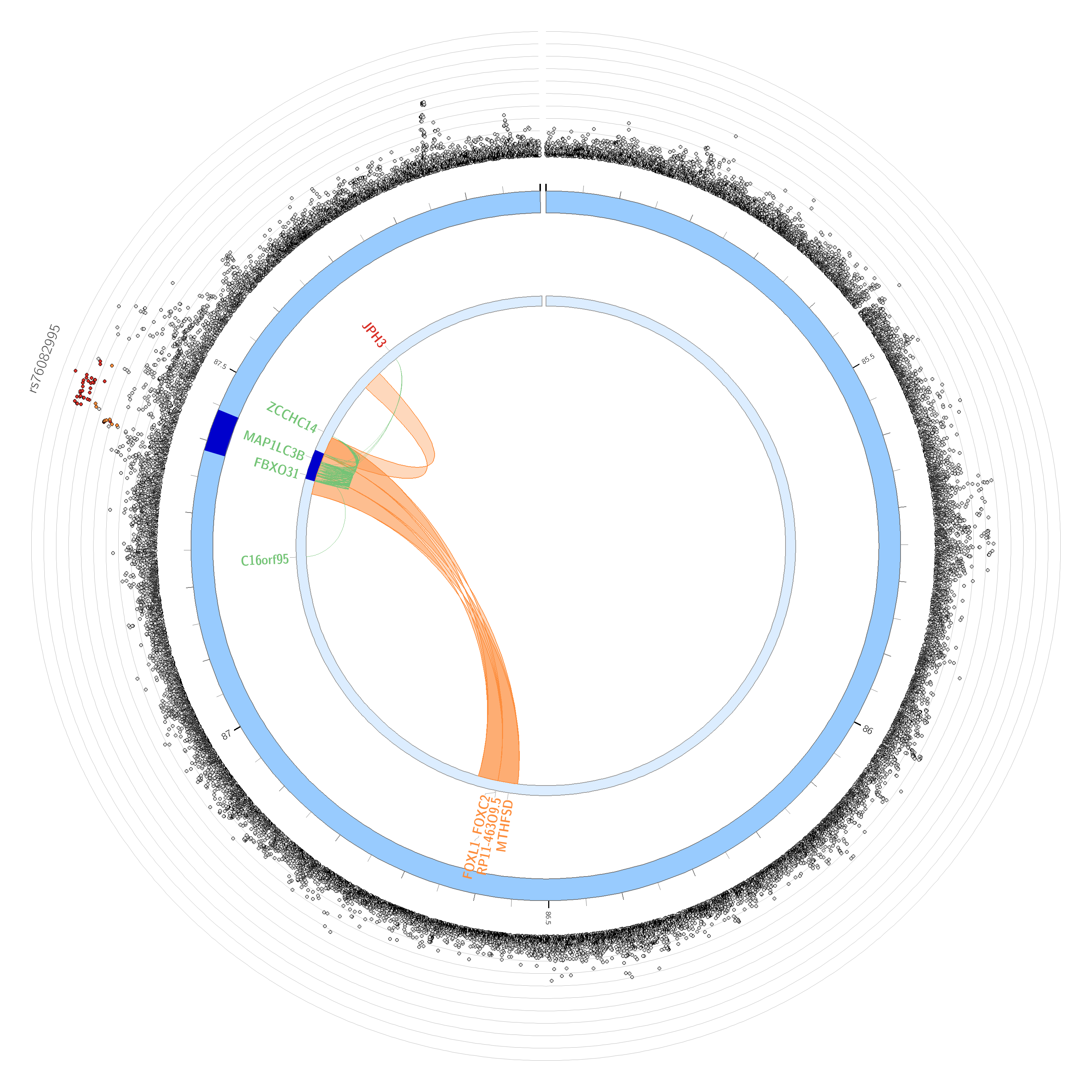

Supplement: Supplementary file 19 — Supplementary Figure 1O CHR16 [file 41380_2019_387_MOESM19_ESM.png]

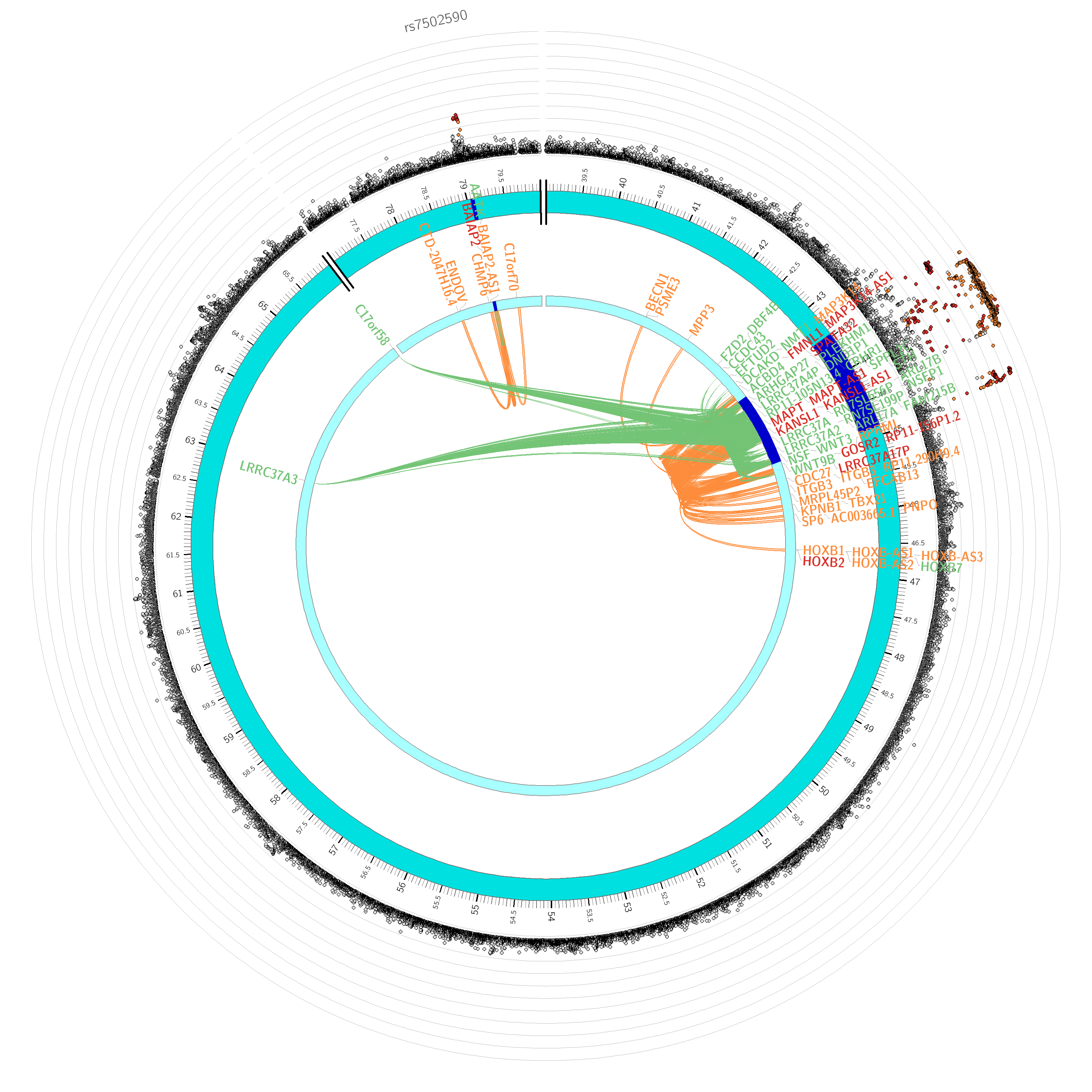

Supplement: Supplementary file 20 — Supplementary Figure 1P CHR17 [file 41380_2019_387_MOESM20_ESM.png]

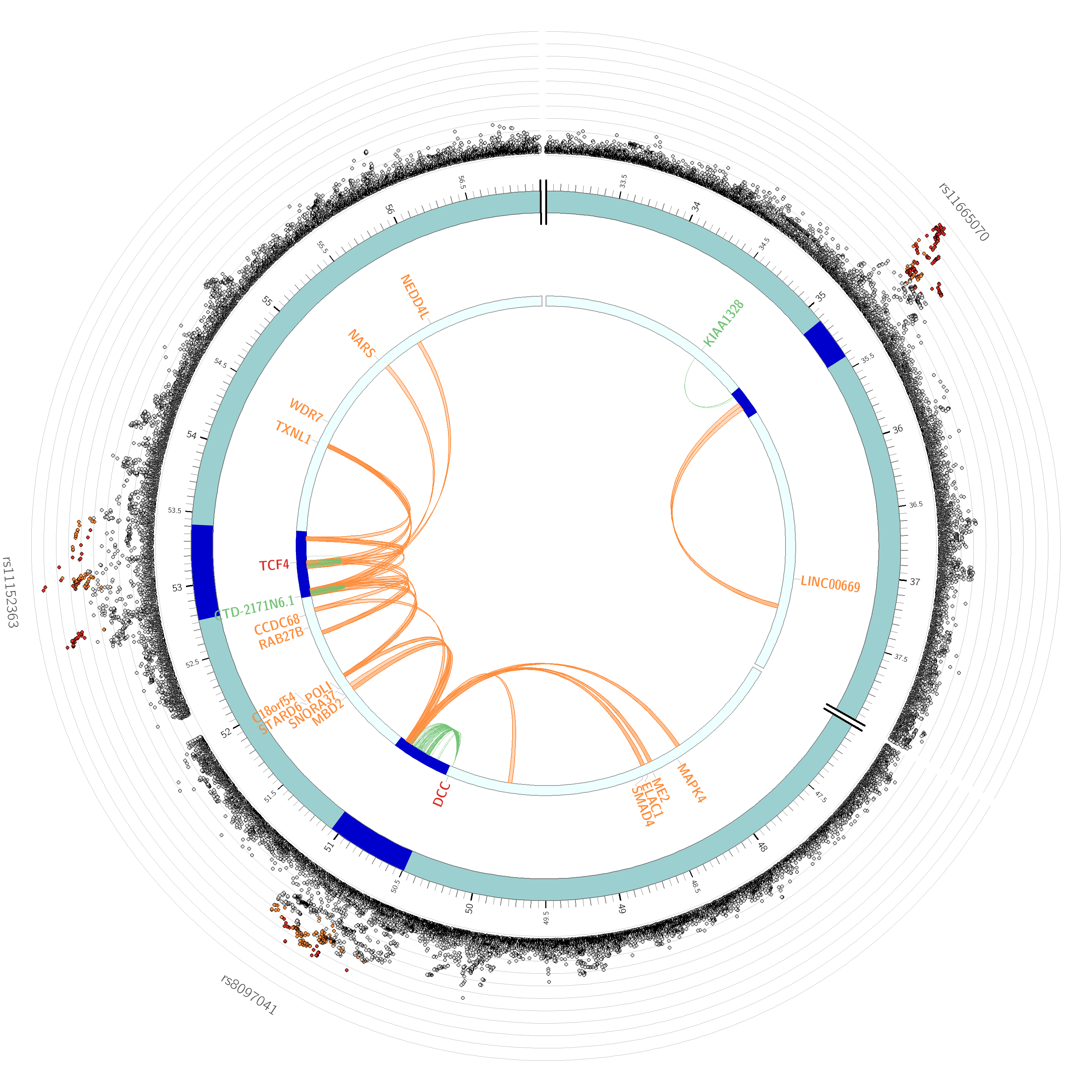

Supplement: Supplementary file 21 — Supplementary Figure 1Q CHR18 [file 41380_2019_387_MOESM21_ESM.png]

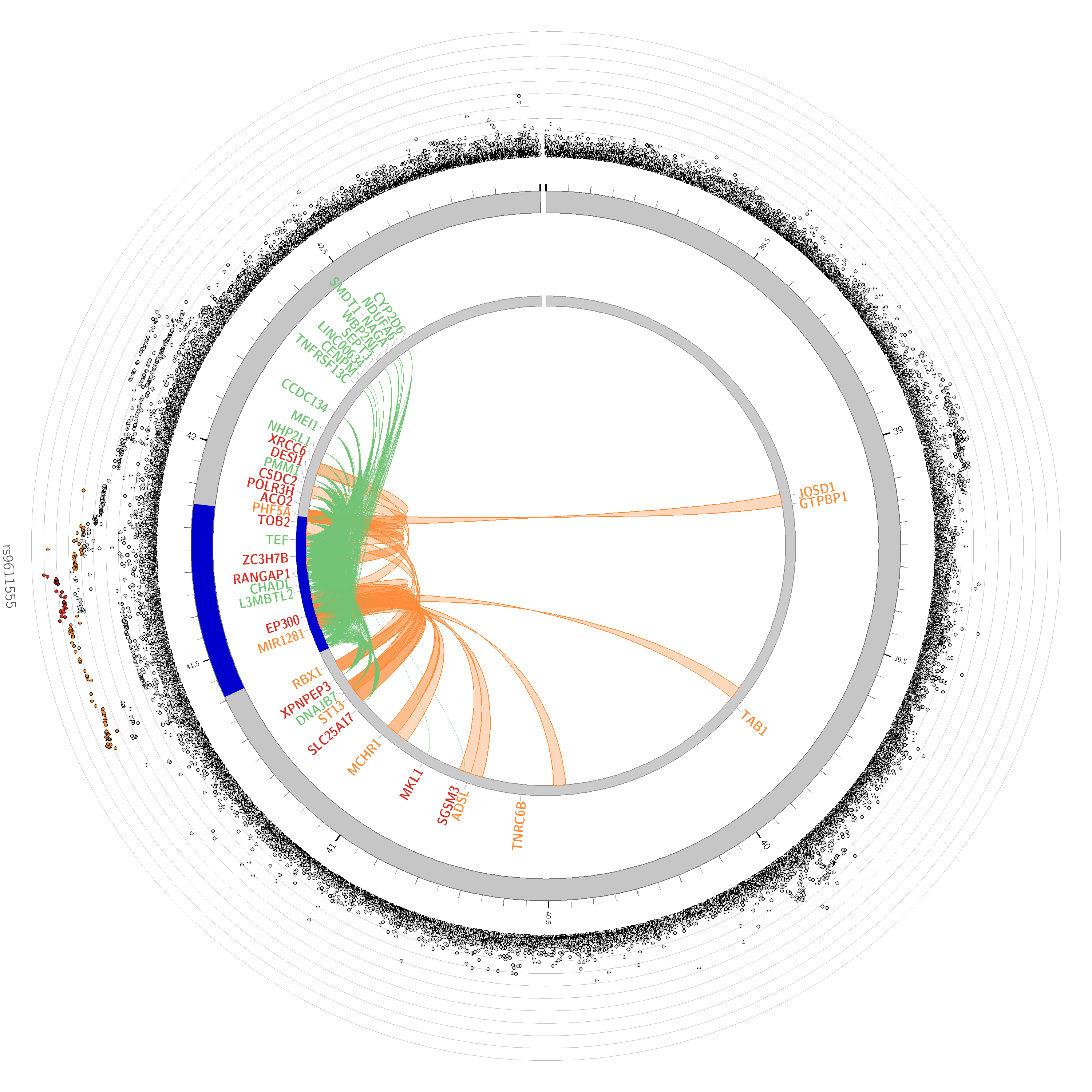

Supplement: Supplementary file 22 — Supplementary Figure 1R CHR22 [file 41380_2019_387_MOESM22_ESM.png]

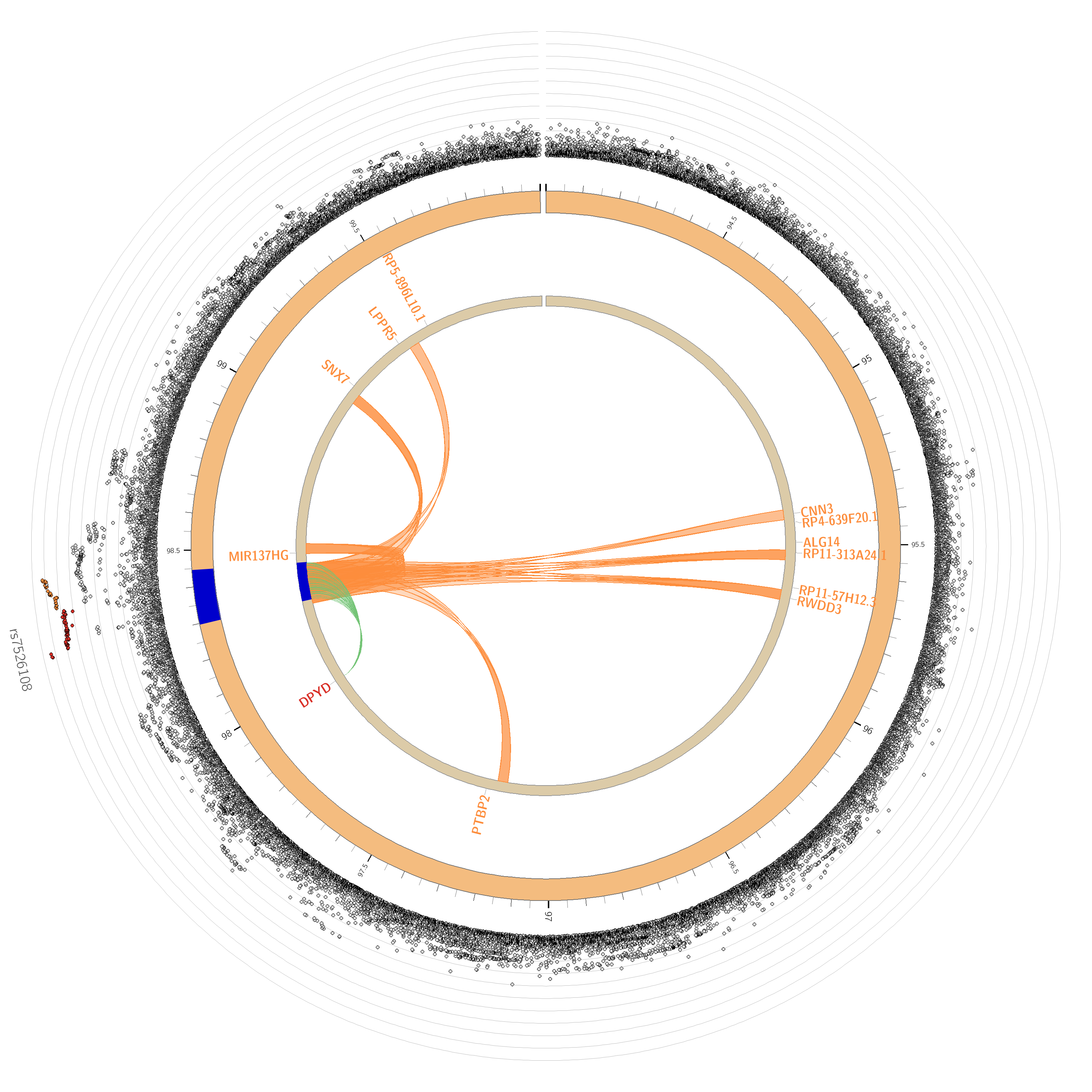

Supplement: Supplementary file 23 — Supplementary Figure 2A CHR1 [file 41380_2019_387_MOESM23_ESM.png]

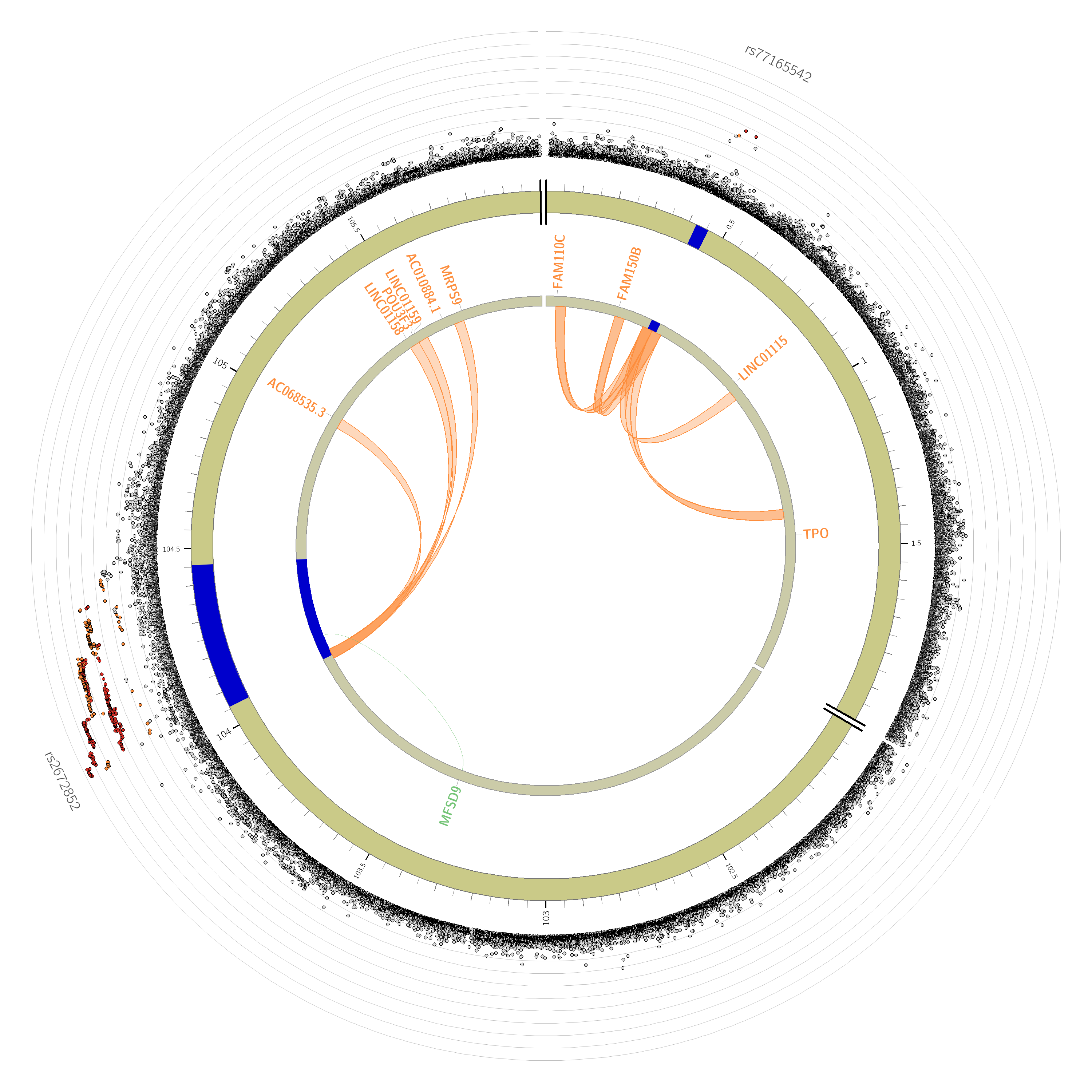

Supplement: Supplementary file 24 — Supplementary Figure 2B CHR2 [file 41380_2019_387_MOESM24_ESM.png]

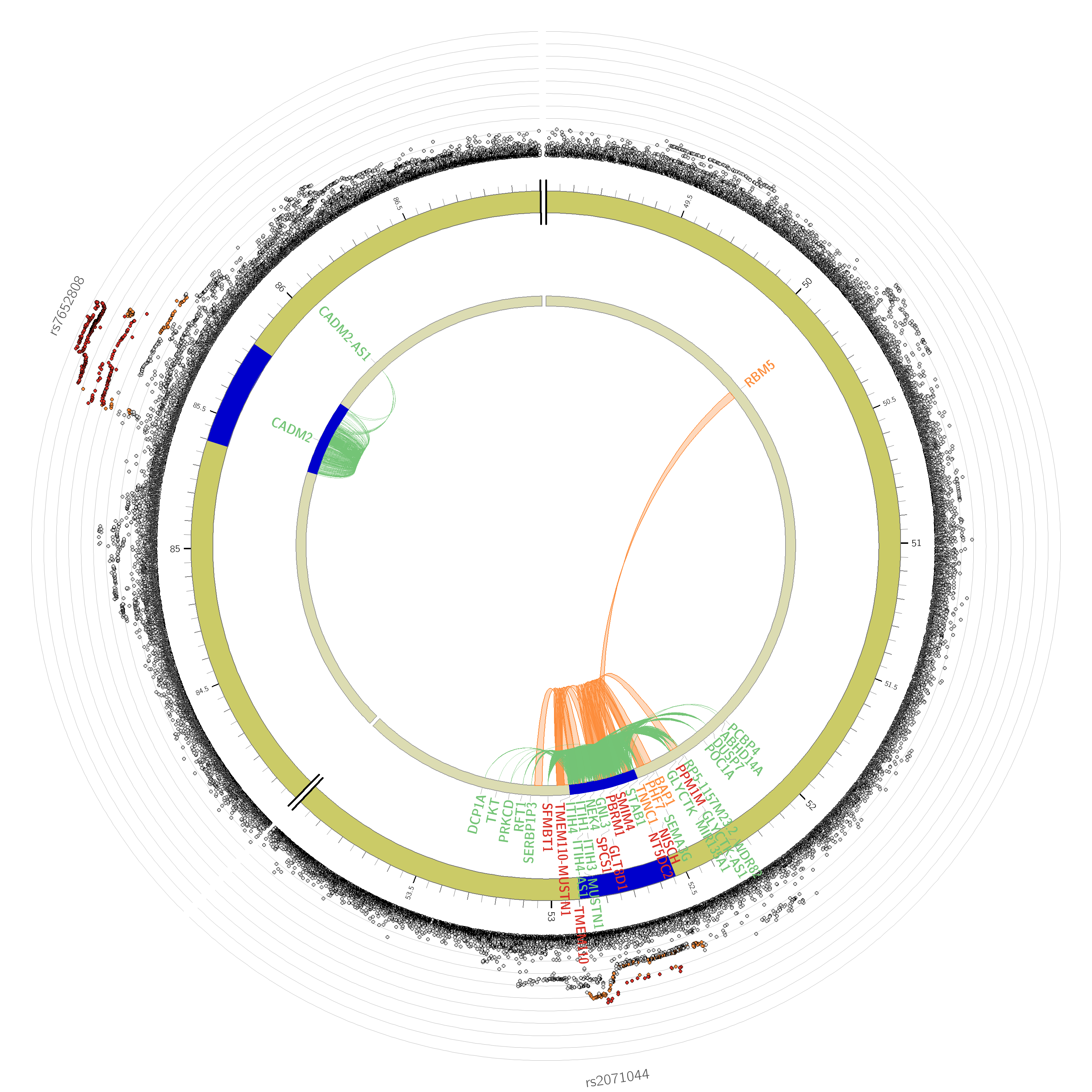

Supplement: Supplementary file 25 — Supplementary Figure 2C CHR3 [file 41380_2019_387_MOESM25_ESM.png]

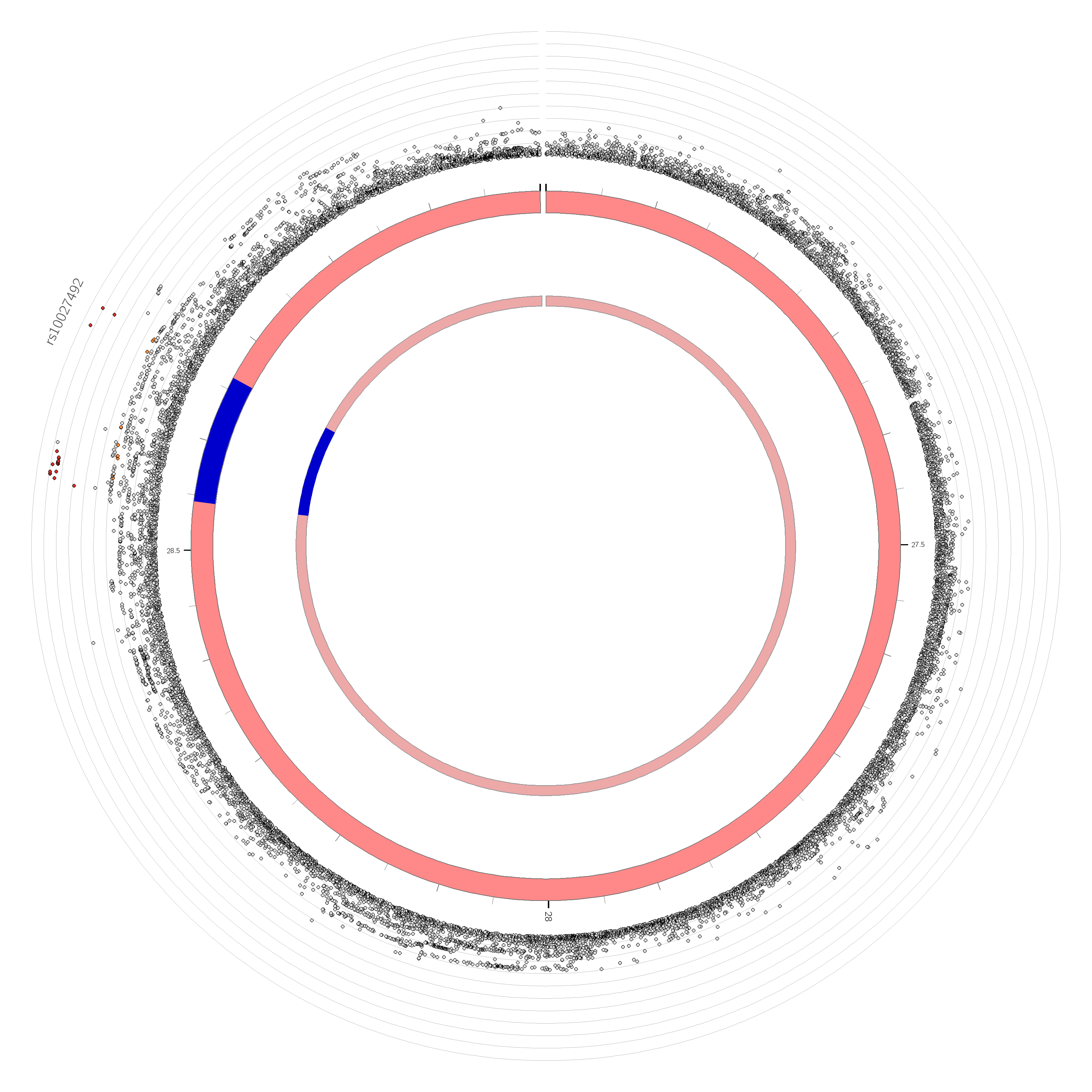

Supplement: Supplementary file 26 — Supplementary Figure 2D CHR4 [file 41380_2019_387_MOESM26_ESM.png]

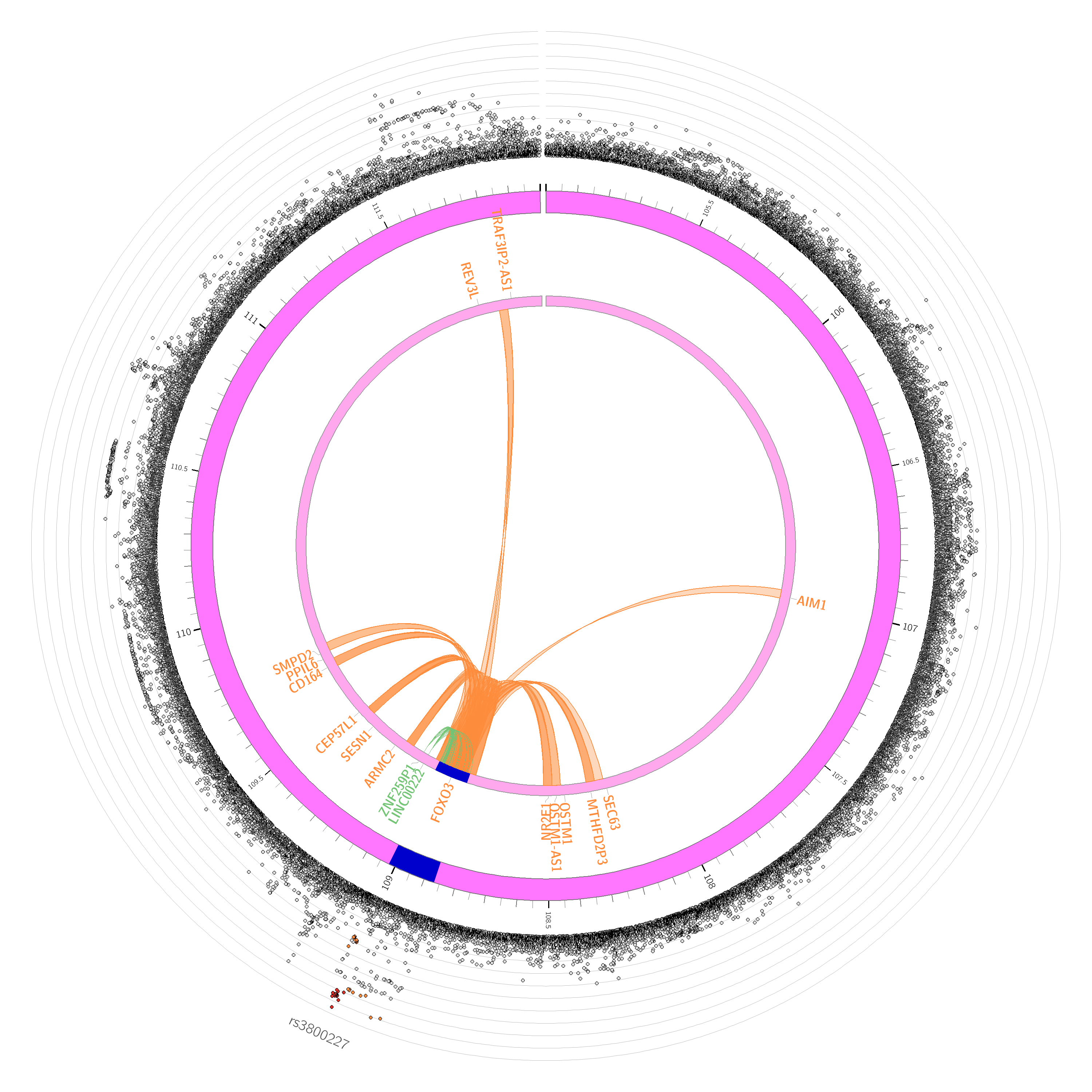

Supplement: Supplementary file 27 — Supplementary Figure 2E CHR6 [file 41380_2019_387_MOESM27_ESM.png]

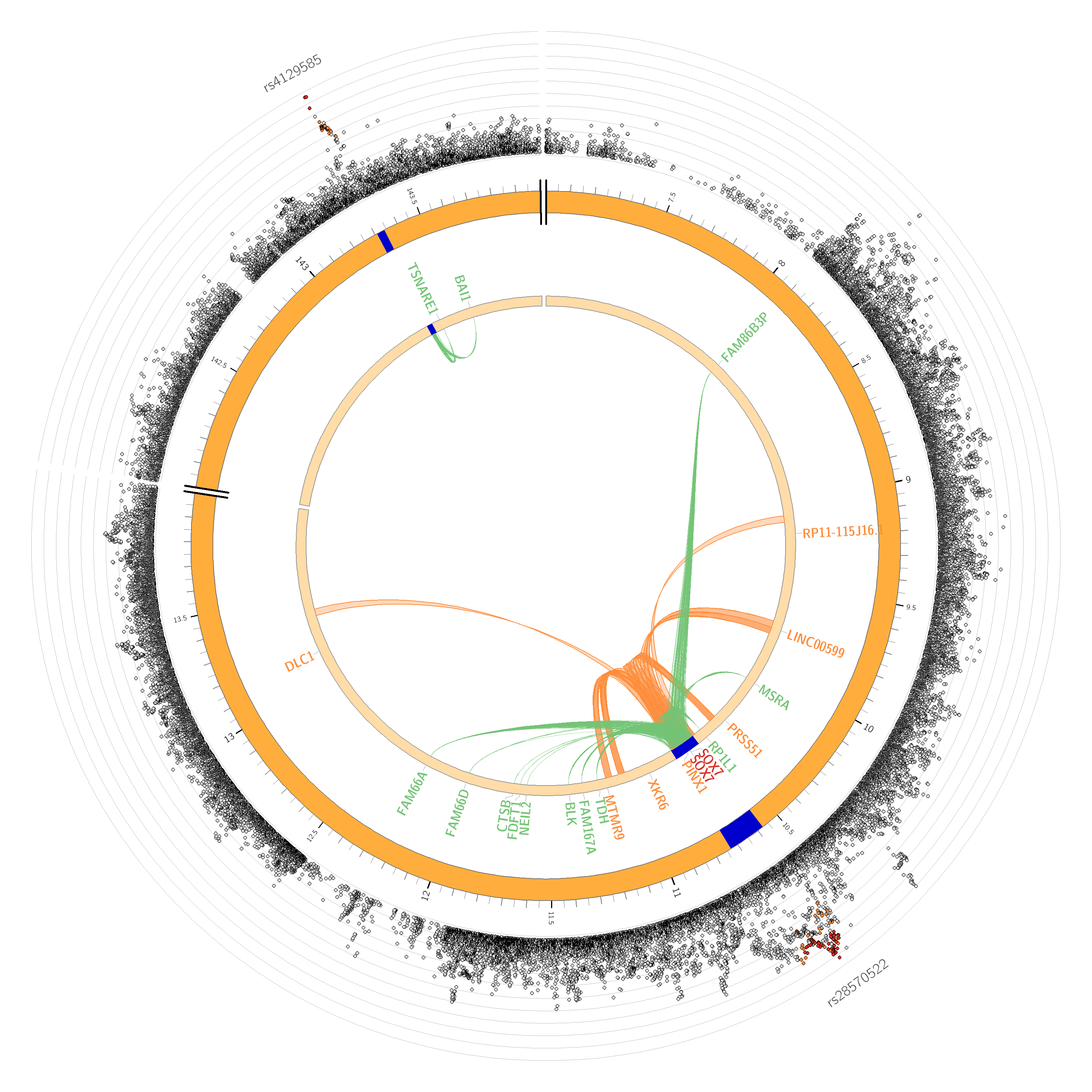

Supplement: Supplementary file 28 — Supplementary Figure 2F CHR8 [file 41380_2019_387_MOESM28_ESM.png]

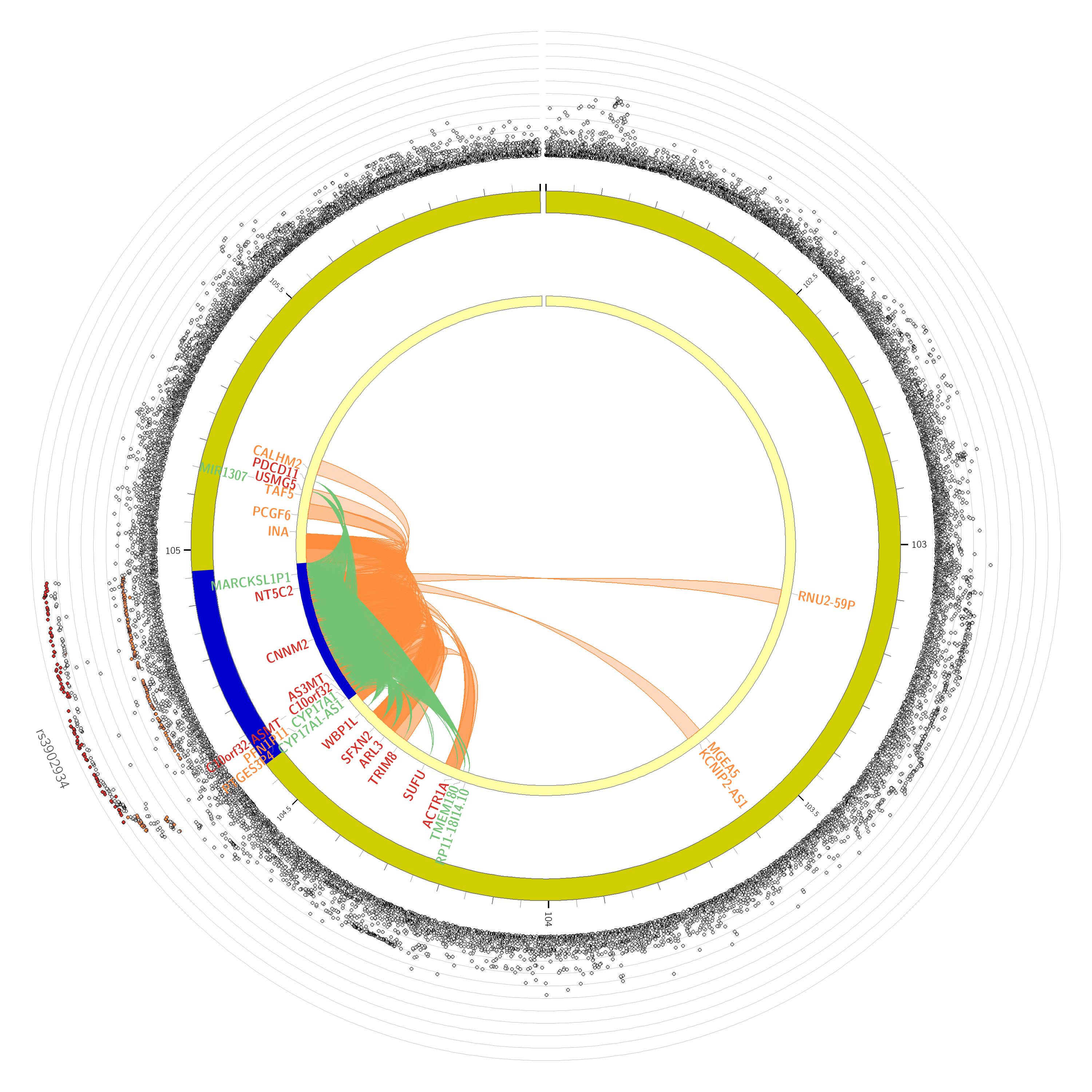

Supplement: Supplementary file 29 — Supplementary Figure 2G CHR10 [file 41380_2019_387_MOESM29_ESM.png]

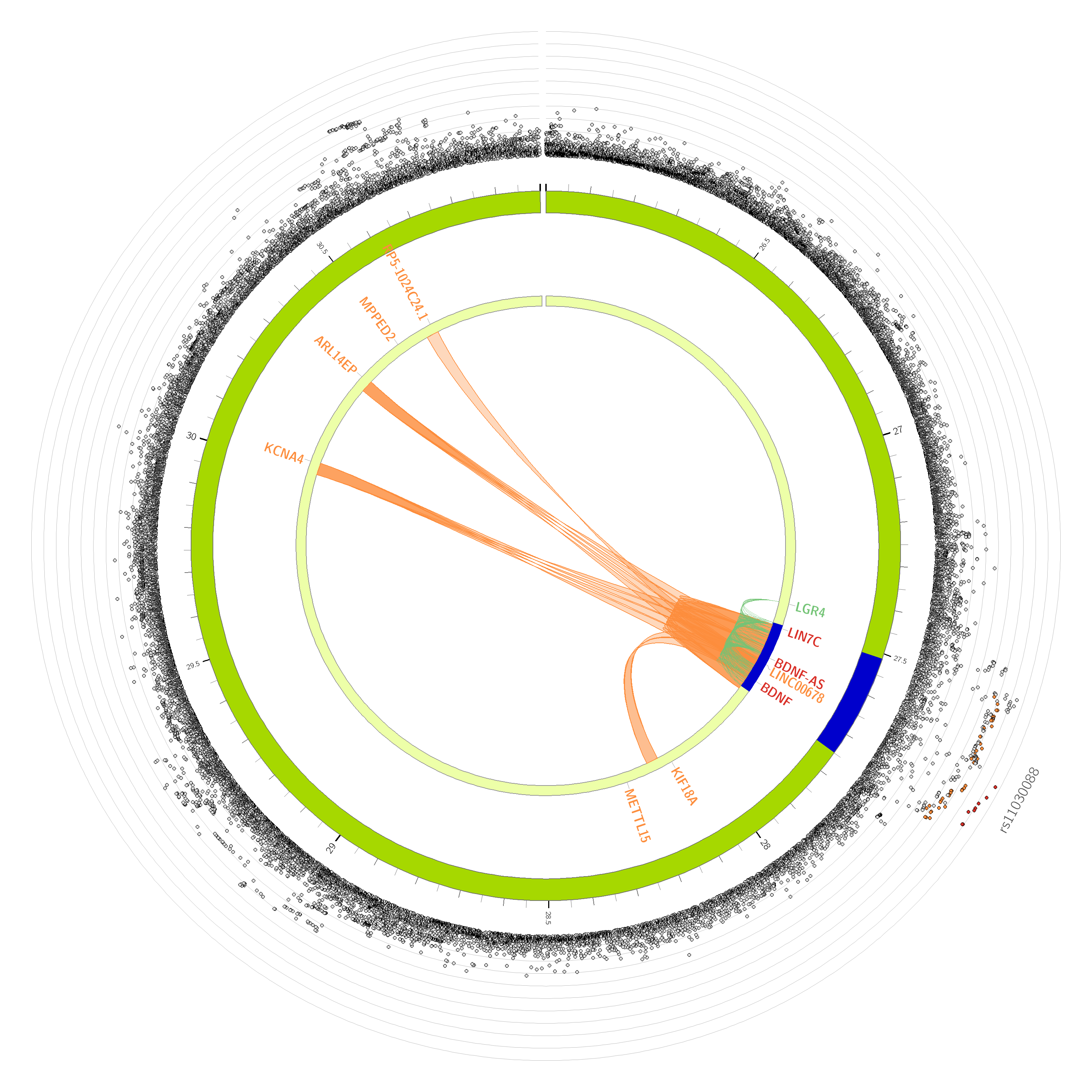

Supplement: Supplementary file 30 — Supplementary Figure 2H CHR11 [file 41380_2019_387_MOESM30_ESM.png]

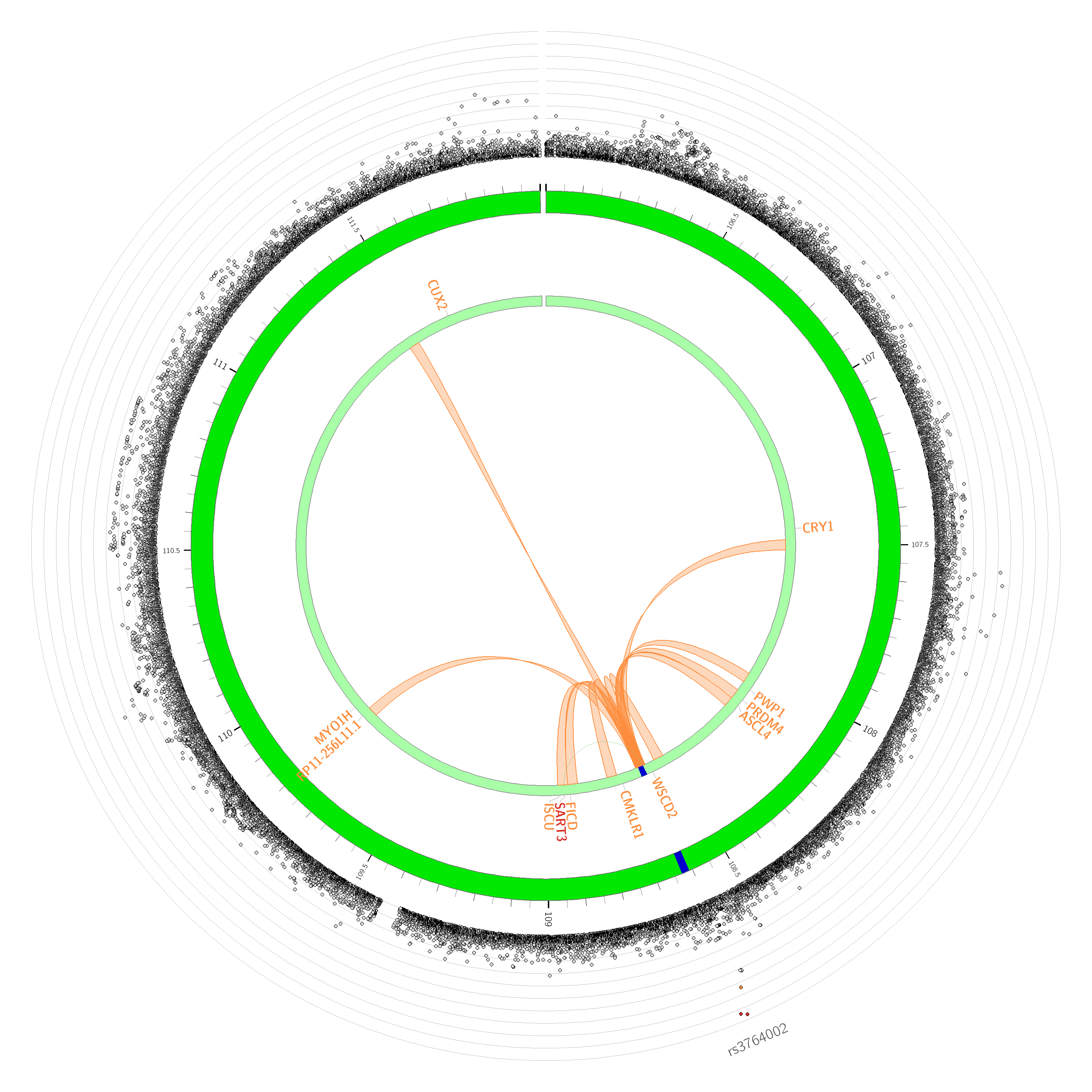

Supplement: Supplementary file 31 — Supplementary Figure 2I CHR12 [file 41380_2019_387_MOESM31_ESM.png]

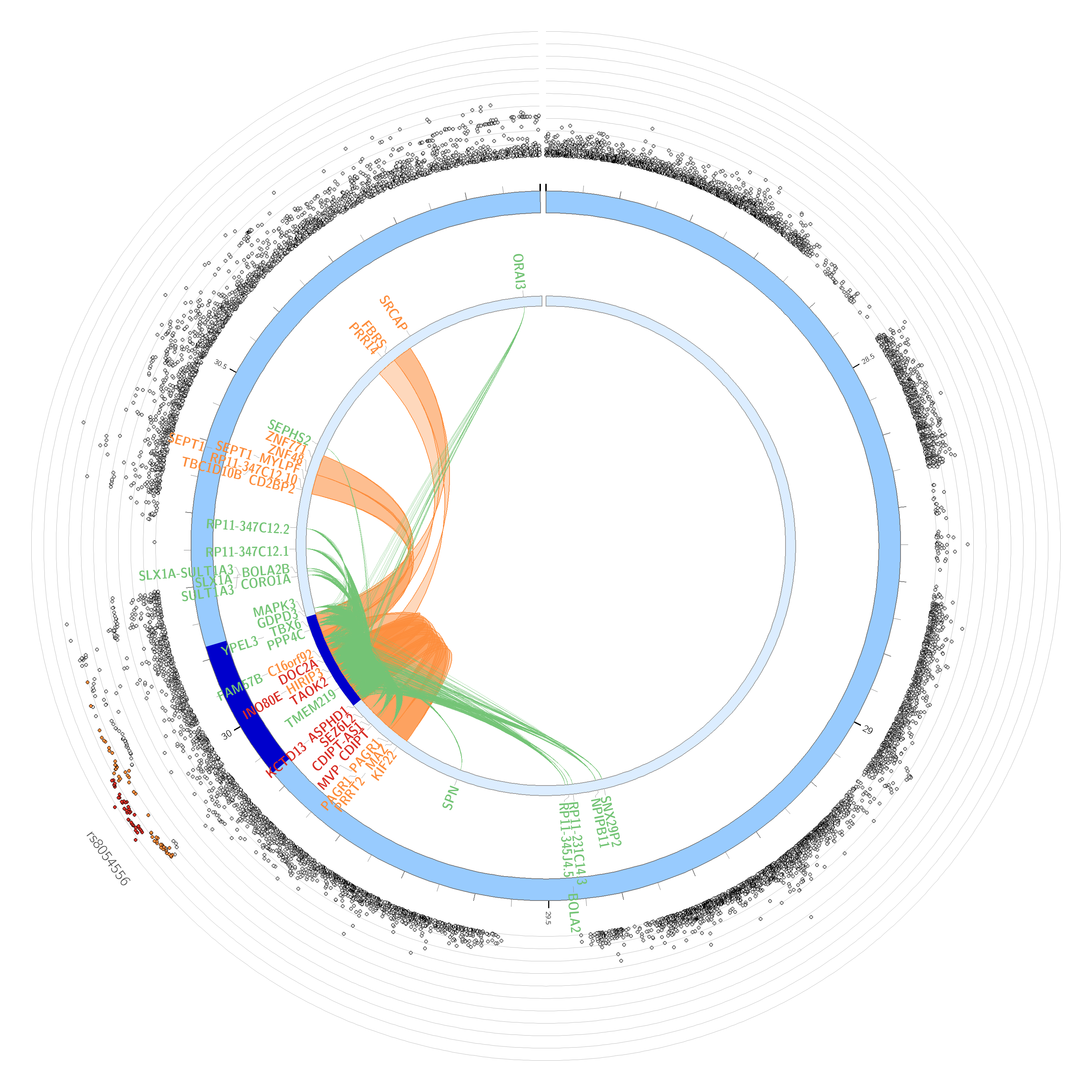

Supplement: Supplementary file 32 — Supplementary Figure 2J CHR16 [file 41380_2019_387_MOESM32_ESM.png]

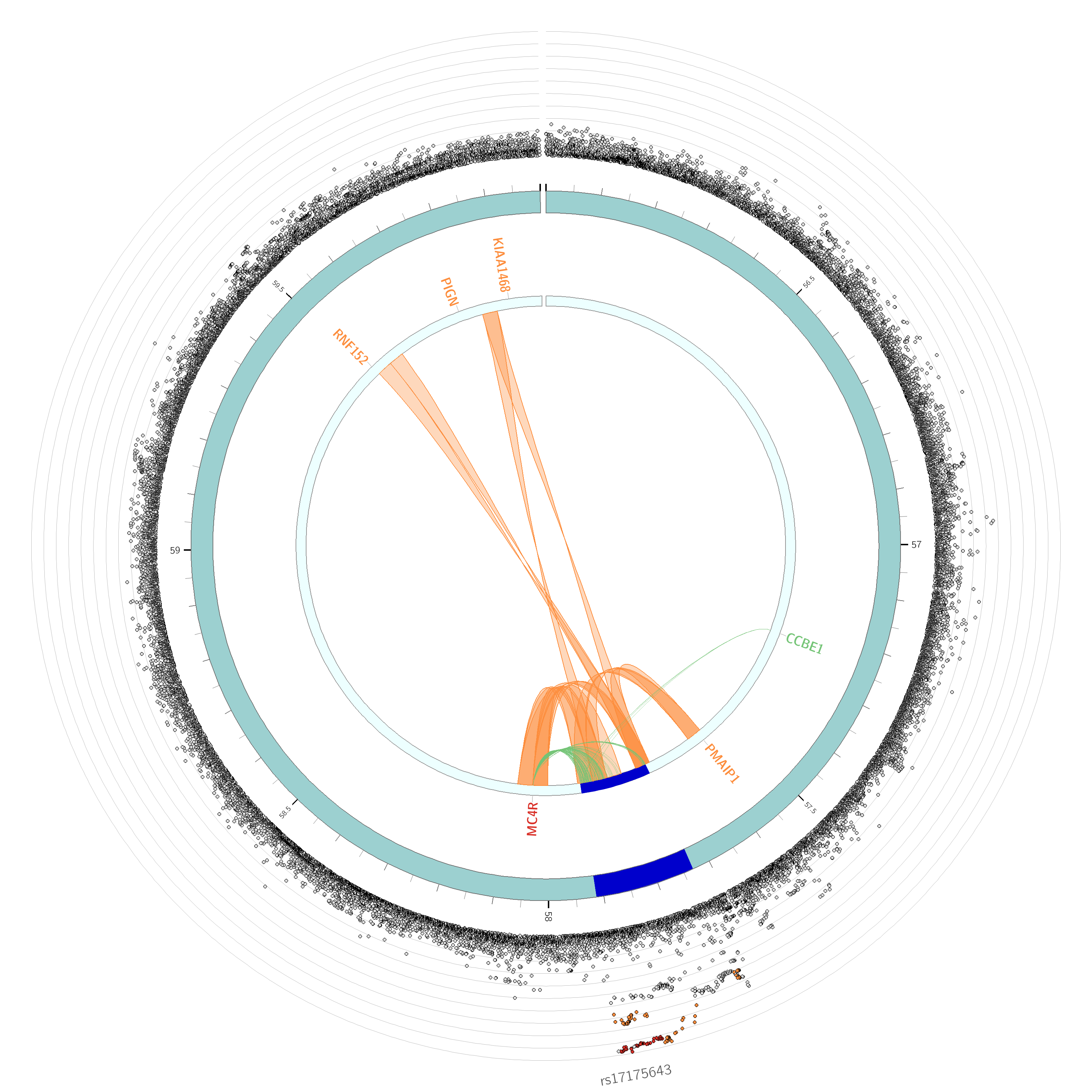

Supplement: Supplementary file 33 — Supplementary Figure 2K CHR18 [file 41380_2019_387_MOESM33_ESM.png]

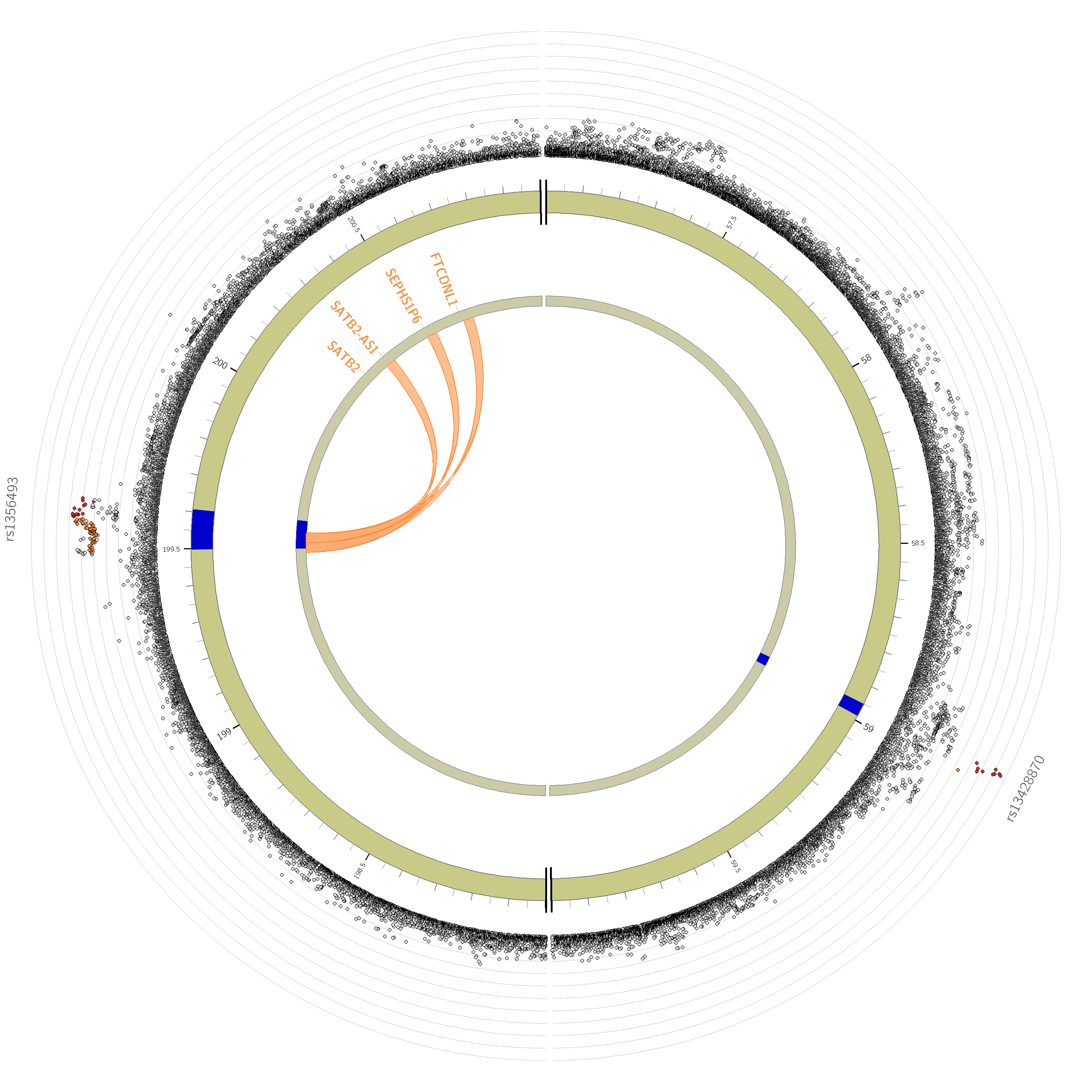

Supplement: Supplementary file 34 — Supplementary Figure 3A CHR2 [file 41380_2019_387_MOESM34_ESM.png]

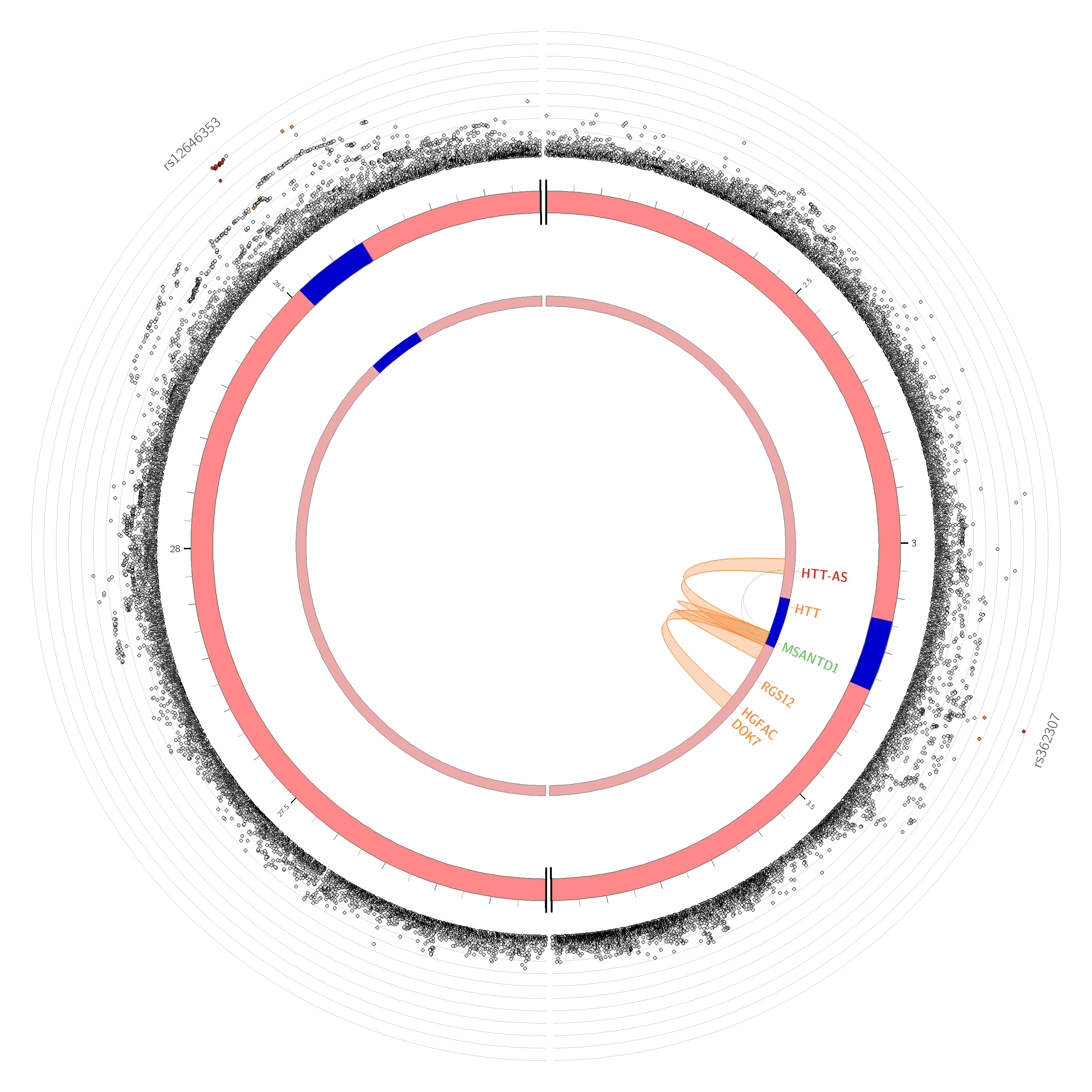

Supplement: Supplementary file 35 — Supplementary Figure 3B CHR4 [file 41380_2019_387_MOESM35_ESM.png]

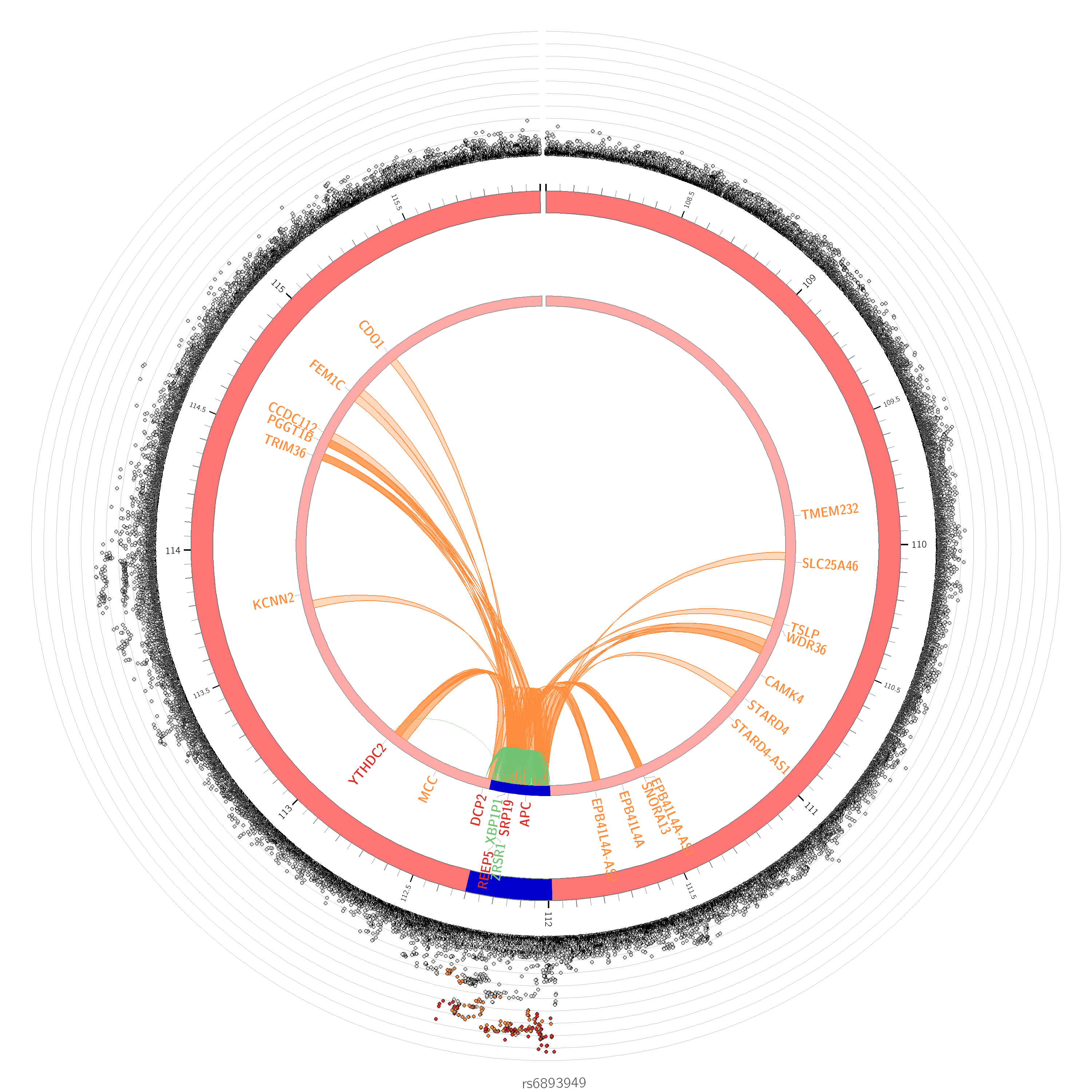

Supplement: Supplementary file 36 — Supplementary Figure 3C CHR5 [file 41380_2019_387_MOESM36_ESM.png]

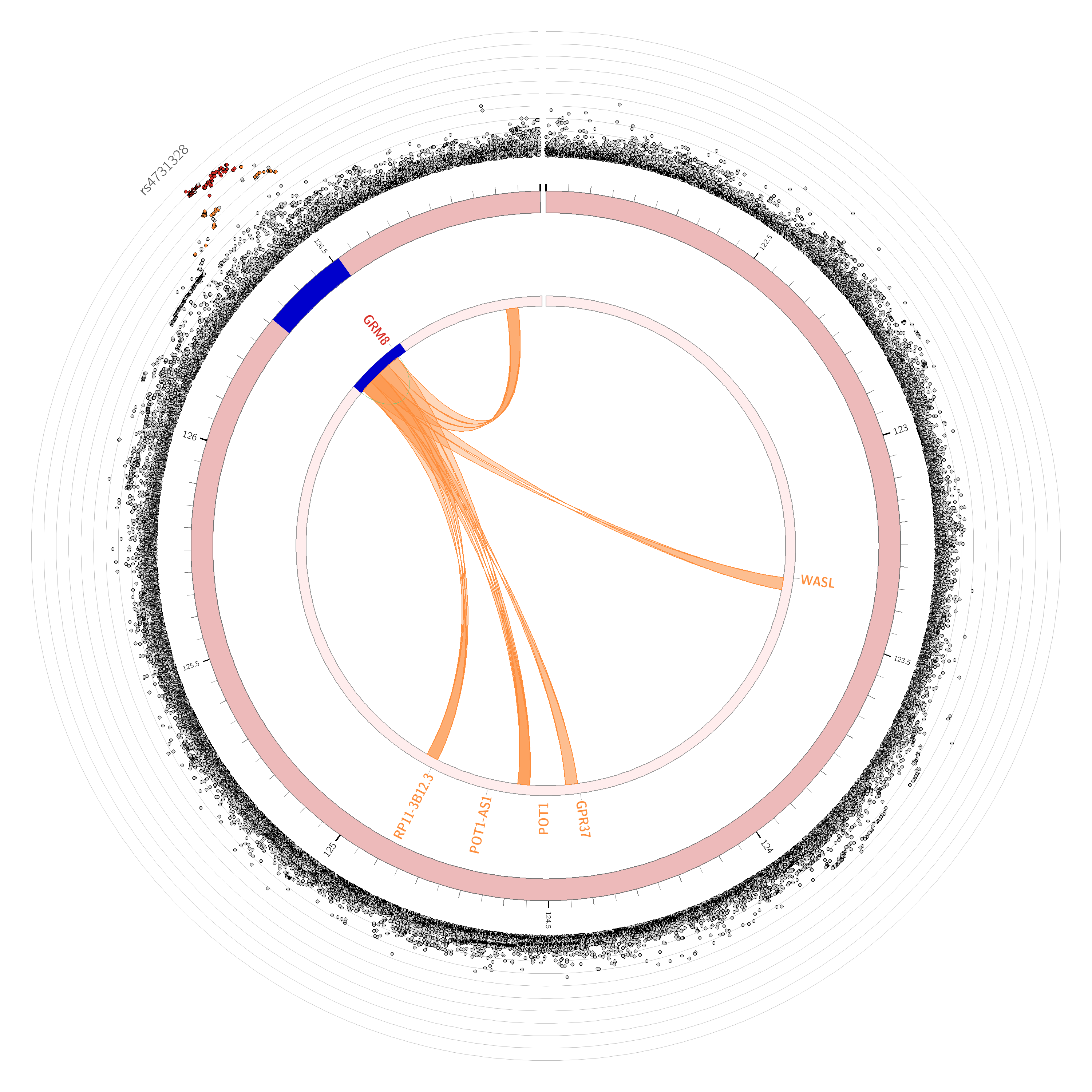

Supplement: Supplementary file 37 — Supplementary Figure 3D CHR7 [file 41380_2019_387_MOESM37_ESM.png]

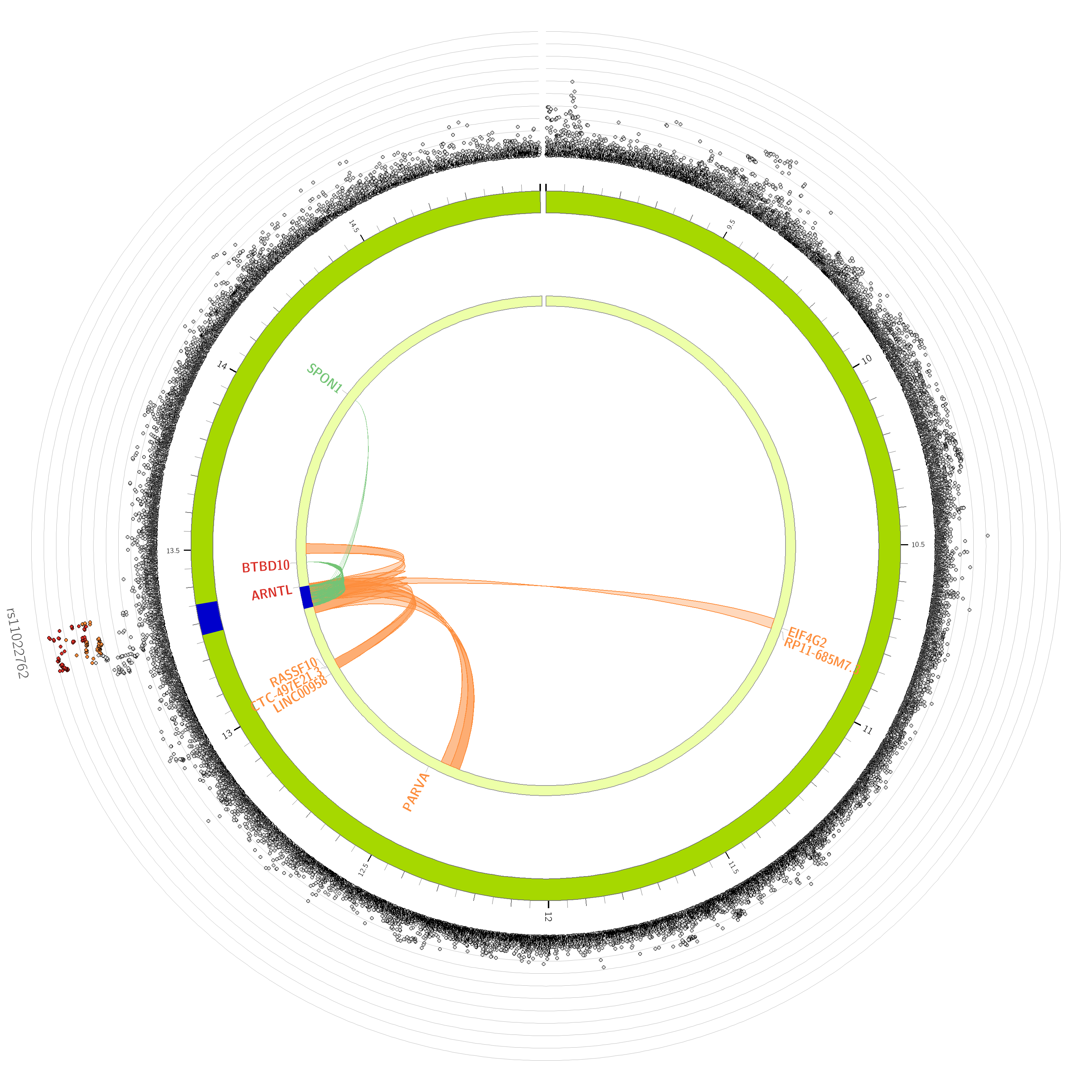

Supplement: Supplementary file 38 — Supplementary Figure 3E CHR11 [file 41380_2019_387_MOESM38_ESM.png]

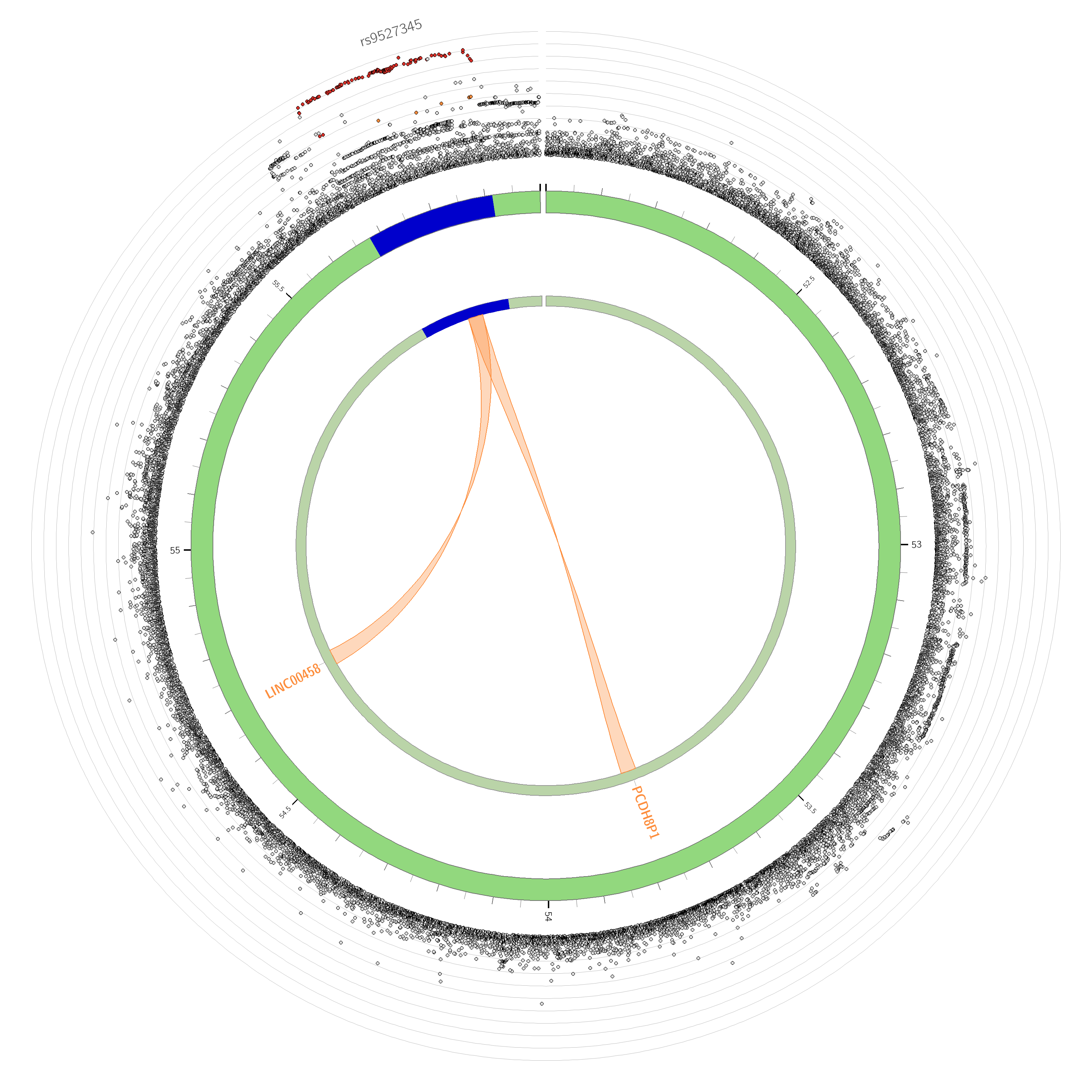

Supplement: Supplementary file 39 — Supplementary Figure 3F CHR13 [file 41380_2019_387_MOESM39_ESM.png]

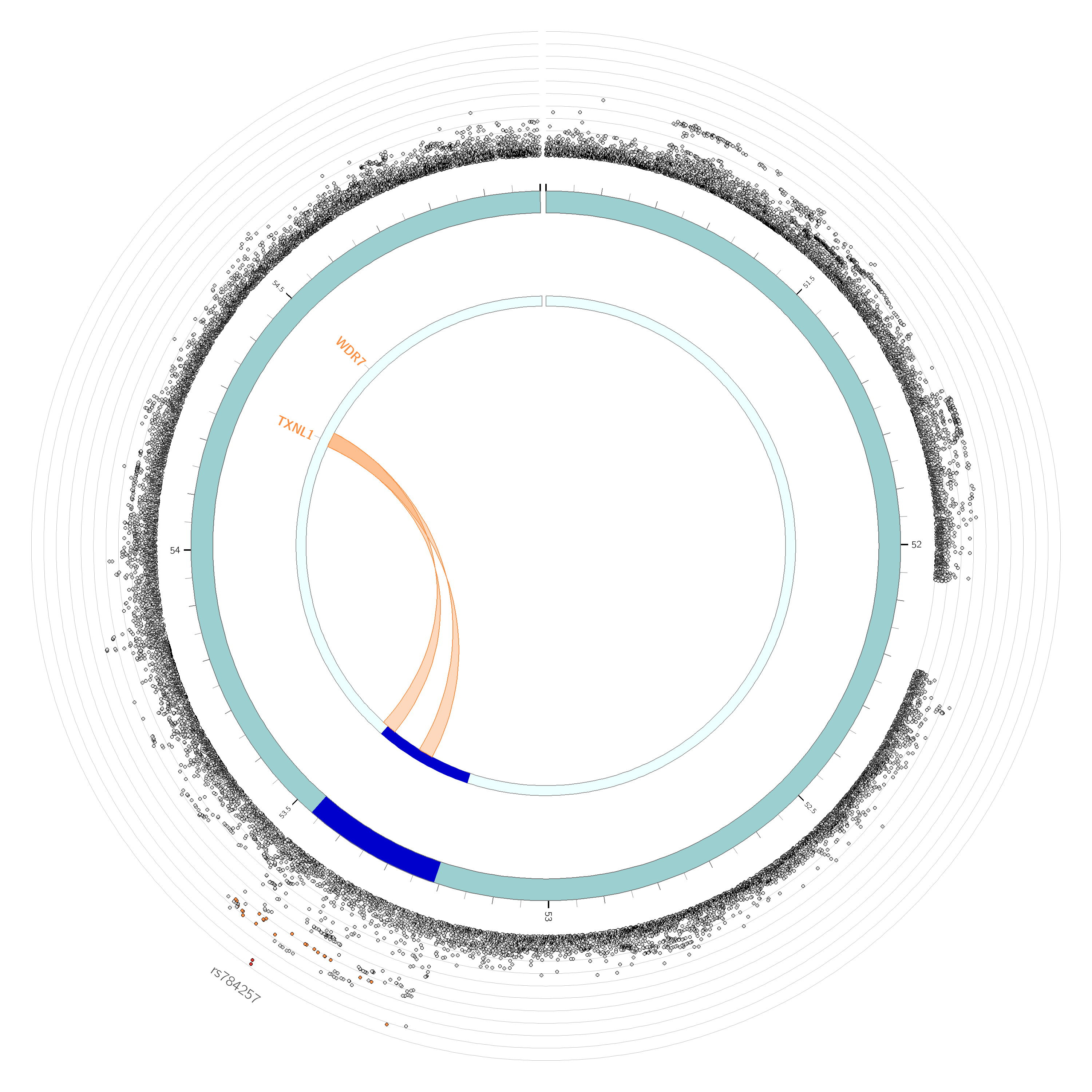

Supplement: Supplementary file 40 — Supplementary Figure 3G CHR18 [file 41380_2019_387_MOESM40_ESM.png]

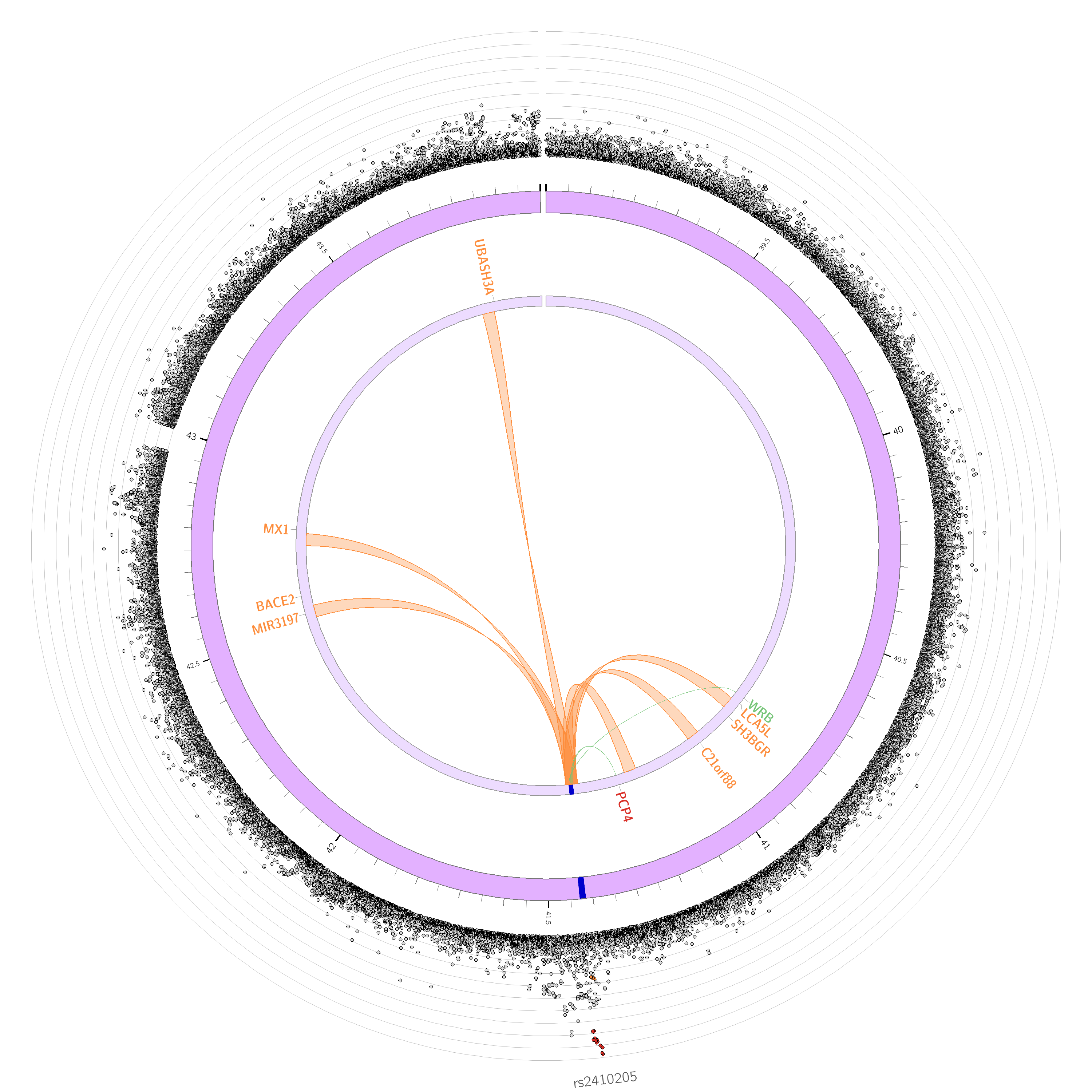

Supplement: Supplementary file 41 — Supplementary Figure 3H CHR21 [file 41380_2019_387_MOESM41_ESM.png]
